# Keratin 18 functions as a lactyltransferase to trigger necroptosis in diabetic kidney disease by modulating Fas transcription

**DOI:** 10.1038/s12276-026-01737-9

**Published:** 2026-06-12

**Authors:** Qiao Zhao, Xu Liu, Yangliang Yang, Fufen Meng, Zhipeng Meng, Minmin Zhu

**Affiliations:** 1 Department of Anesthesiology, Shanghai Pudong Hospital, Fudan University Pudong Medical Center, Shanghai, China; 2 Department of Anesthesiology, Xinjiang Medical University Affiliated Cancer Hospital, Urumqi, Xinjiang China; 3 Department of Anesthesiology, Huzhou Central Hospital, Affiliated Central Hospital of HuZhou University, Huzhou, Zhejiang China

**Keywords:** Diabetes complications, Diabetes complications

## Abstract

Diabetic kidney disease (DKD) is a type of chronic renal injury induced by diabetes mellitus and is characterized by persistent proteinuria and progressive decreases in the glomerular filtration rate. Recent studies have highlighted the significance of histone lactylation and necroptosis in the pathogenesis of DKD. We explored the mechanisms by which lactate-induced histone H3 lysine-18 lactylation (H3K18la) and H3K27la promote necroptosis in DKD. Lactate-induced H3K18la and H3K27la modulated Fas transcription, contributing to necroptosis and DKD progression. Moreover, keratin 18 (KRT18), identified as a lactyltransferase, regulated H3K18la and H3K27la levels, subsequently inducing Fas transcription and necroptosis. Furthermore, ginsenoside Rc (gRc) inhibited KRT18 lactyltransferase activity by competing for the lactate binding site in KRT18. Notably, gRc treatment reduced the KRT18, H3K18la, H3K27la, and Fas levels and alleviated necroptosis and renal dysfunction in DKD models. In conclusion, KRT18 functions as a lactyltransferase to induce Fas transcription and necroptosis. Moreover, inhibiting KRT18-mediated histone lactylation via gRc is a potential strategy for treating DKD.

## KRT18 mediates necroptosis through histone lactylation

Diabetic kidney disease (DKD) is a major complication of diabetes, leading to end-stage renal failure owing to subtle early symptoms and lack of specific treatments. This study explores the role of lactate-induced histone lactylation in DKD, focussing on the enzymes regulating this process. Researchers used DKD mouse models and cellular experiments to investigate how lactate accumulation affects histone modifications, specifically lactate-induced histone H3 lysine-18 lactylation (H3K18la) and H3K27la, which are linked to necroptosis, a form of programmed cell death. The study found that inhibiting lactate production with lactate dehydrogenase A inhibitors reduced these histone modifications, necroptosis, and renal injury. Furthermore, KRT18 was identified as a lactyltransferase that modifies histones, promoting Fas transcription and necroptosis. The compound ginsenoside Rc was shown to inhibit KRT18 activity, reducing necroptosis and renal damage, suggesting a potential therapeutic strategy for DKD. Future research could explore other lactylation pathways and therapeutic targets in DKD.

This summary was initially drafted using artificial intelligence, then revised and fact-checked by the author.

## Introduction

Diabetic kidney disease (DKD) is a chronic kidney injury induced by diabetes mellitus and is characterized primarily by persistent proteinuria and a progressive decline in the glomerular filtration rate^1,2^. In its advanced stages, DKD leads to end-stage renal failure^3,4^. Clinical features include microalbuminuria, hypertension, edema, and the gradual deterioration of renal function. A major challenge in managing DKD lies in the subtle nature of its early symptoms and the absence of specific therapeutic strategies for reversing the condition^5^. Although current interventions, such as glycemic control and antihypertensive therapy, can slow disease progression, they are insufficient to fully prevent the development of end-stage renal disease^6^. Therefore, exploration of innovative therapeutic approaches for DKD is urgently needed.

In DKD, the synergistic interaction of multiple forms of cell death collectively exacerbates renal injury^7^. Notably, necroptosis, which is mediated by the receptor-interacting kinase 1 (RIPK1)/RIPK3/mixed lineage kinase domain-like protein (MLKL) signaling pathway, promotes fibrosis and represents one of the core pathological mechanisms underlying DKD^8,9^. The upregulation of the Fas/Fas ligand (FasL) signaling pathway has been reported to trigger RIPK1/RIPK3/MLKL-dependent necroptosis in renal tubular cells^10^ and adipocytes^11^. Two studies^10,11^ have revealed the key role of Fas/FasL as a “molecular switch“ for necroptosis, which may provide new potential targets for the treatment of metabolic diseases such as DKD.

Multiple glucose metabolic products, including lactate, have critical roles in the development of diabetes-related complications^12^. Excessive lactate accumulation has been implicated in the pathogenesis of DKD^13^. However, the precise mechanisms by which lactate drives the progression of DKD remain to be elucidated. Epigenetic modifications have been established as key regulators of diabetes-associated complications^14^. Our prior research revealed that both histone methylation^15,16^ and histone acetylation^17^ contribute to the onset and progression of DKD. Recently, histone H3K18 lactylation (H3K18la) was identified as a tissue-specific active enhancer^18^. Furthermore, emerging evidence suggests that lactate-mediated histone lactylation, a novel epigenetic modification, is involved in the pathophysiology of diabetes-related complications, including DKD^19,20^. Nevertheless, the role of histone lactylation in triggering necroptosis in renal tissue cells remains poorly understood.

Lactylation regulatory proteins have essential roles in mediating the transcription of specific genes through the modulation of histone lactylation. HDAC1-3 function as “erasers” by removing histone lactylation marks^21^, whereas alanyl-tRNA synthetase 1 (AARS1) and p300 both act as “writers” that catalyze histone lactylation^22,23^. Furthermore, Brg1 has been identified as a “reader” that recognizes histone lactylation signals and facilitates transcriptional regulation^24^. In addition, some of these regulatory proteins have been confirmed to be involved in the pathological process of DKD and are regarded as potential therapeutic targets^25–27^. However, whether specific lactylation regulatory proteins contribute to DKD remains incompletely characterized. Identification and functional validation of such proteins are therefore essential for identifying novel therapeutic targets for DKD.

Keratin 18 (KRT18), a type I cytokeratin and a member of the intermediate filament family, serves as an essential component of the cytoskeleton^28^. This protein is involved in multiple cellular processes, including resistance to external stress and the maintenance of cytoplasmic and mitochondrial structural integrity^29^. Early studies revealed that KRT18 is expressed in endothelial and epithelial cells^30^. Recent findings indicate that KRT18 exerts its biological functions by modulating the transcription of genes^31^. However, the underlying mechanism remains unclear and deserves further exploration.

In this study, we aimed to elucidate the role of lactate-induced histone lactylation in the pathogenesis of DKD and to investigate the underlying molecular mechanisms involved. Moreover, we aimed to systematically identify the specific lactylation-regulating enzymes involved in this process. More importantly, targeted therapeutic strategies aimed at modulating this epigenetic pathway to prevent or alleviate renal dysfunction in patients with DKD will be explored.

## Materials and methods

### Subjects

This study was conducted in strict adherence to the principles established in the Declaration of Helsinki, and ethical approval was obtained from the Ethics Committee of Huzhou Central Hospital (Ethical Approval Code: 202405039-01). Written informed consent was obtained from all participants with a diagnosis of DKD. Participants who presented with severe hepatic dysfunction, non-diabetic renal insufficiency, cerebrovascular incidents, malignant neoplasms, immune-related disorders, peripheral arterial diseases, or additional cardiovascular comorbidities were excluded from the analysis. The estimated glomerular filtration rate (eGFR) of the participants was determined using the formula established by the Chronic Kidney Disease Epidemiology Collaboration, and the severity of DKD was classified as stage 2, 3, or 4 according to the eGFR measurements.

### Mouse model of DKD

This research received ethical approval from the Shanghai Pudong Hospital Laboratory Animal Welfare and Ethics Committee (Ethical Approval Code: KCZX20250303-002). MLKL heterozygous mice (MLKL^+/^^−^) and KRT18 heterozygous mice (KRT18^+/^^−^) on a C57BL/6 genetic background were acquired from Jicui Biotechnology Limited (Jiangsu, China). Age-matched male wild-type, MLKL homozygous knockout (MLKL^−/−^), and KRT18 homozygous knockout (KRT18^−/−^) littermates bred in our facility were used at 2 months of age. The animals were maintained in a temperature-regulated environment (22–25 °C) on a 12-h light/dark cycle. The DKD (*n* = 8), DKD + MLKL^−/−^ (*n* = 8), and DKD + KRT18^−/−^ (*n* = 8) groups were established using a previously described method^16^. Briefly, the mice underwent unilateral nephrectomy while they were anesthetized following an intraperitoneal injection of 80 mg/kg ketamine and 5 mg/kg xylazine. Following the procedure, the mice were returned to the care facility for 15 weeks. Two weeks after nephrectomy, the mice that received an intraperitoneal injection of citrate buffer (0.1 M, pH 4.5) for 5 consecutive days were assigned to the control group (Con, *n* = 8). Following nephrectomy, animals in the DKD group were maintained on a high-fat, high-sugar diet for 15 weeks. This dietary regimen was supplemented with an intraperitoneal injection of streptozotocin (STZ, 50 mg/kg for 5 consecutive days; HY-13753, MCE, China), which was administered 2 weeks after nephrectomy. To measure blood glucose levels, all mice were fasted for 6 h, after which tail vein blood samples were collected. Model establishment was considered successful for DKD model mice, DKD + MLKL^−/−^ mice, and DKD + KRT18^−/−^ mice whose fasting blood glucose levels exceeded 16.7 mmol/l (ref. ^32^). To inhibit lactate production in DKD model mice, oxamate (OXA, HY-W013032A; MCE) and stiripentol (STP, HY-103392; MCE) were used in this study. OXA was dissolved in saline, whereas STP was dissolved in DMSO (Sigma‒Aldrich, D2650, USA). Mice that received daily intraperitoneal injections of 500 mg/kg OXA during the remaining 12 weeks of the study were assigned to either the OXA group (*n* = 8) or the DKD + OXA group (*n* = 8). The mice that were intraperitoneally injected with 150 mg/kg STP once a day during the final 12 weeks of the study were assigned to either the STP group (*n* = 8) or the DKD + STP group (*n* = 8). To further determine whether lactate-induced H3K18la and H3K27la have important roles in DKD, lactate was used to treat the mice in this study. Mice that were intraperitoneally injected with 1 g/kg lactate (HY-B2227; MCE) once a day after the injection of citrate buffer or STZ were assigned to the lactate group (*n* = 8) or the DKD + lactate group (*n* = 8), respectively. To determine whether ginsenoside Rc (gRc) improves renal dysfunction by inhibiting KRT18-induced H3K18la and H3K27la in DKD model mice, gRc was used to treat DKD model mice in this study. gRc was dissolved in saline. The mice that were intraperitoneally injected with 14 mg/kg gRc (HY-N0042; MCE) once a day during the past 12 weeks of the study were assigned to the gRc group (*n* = 8) and the DKD + gRc group (*n* = 8).

### Hematoxylin and eosin staining, Masson’s trichrome staining, and immunohistochemistry

In the present study, hematoxylin and eosin staining was performed to evaluate the overall renal architecture, with particular attention given to morphological alterations in renal tubules, glomeruli, and the surrounding interstitial tissue. In brief, the paraffin-embedded sections were placed in an oven and maintained at 60 °C for 1–2 h. They were then processed for dewaxing using xylene (10023418; National Pharmaceutical Group, Beijing, China) and ethanol. Nuclear staining was performed with hematoxylin (H3136; Sigma‒Aldrich, St Louis, MO, USA) for ~10 min, followed by cytoplasmic staining with eosin (E4009; Sigma‒Aldrich) for 30 s. Finally, the sections were mounted using neutral balsam (10004160, National Pharmaceutical Group), air-dried at room temperature, and observed under an optical microscope (Nikon Eclipse Ci-L, Nikon). Masson’s trichrome staining and immunohistochemistry (IHC) staining were performed as previously described^16^. The primary antibodies used for the IHC analysis are listed in Supplementary Table 1.

### Immunofluorescence staining

Following the dewaxing and hydration of the paraffin-embedded mouse kidney sections, the sections were treated with 100 μl of hydrogen peroxide blocking solution at room temperature for 10 min to inhibit endogenous peroxidase activity. Antigen retrieval was subsequently performed by heating the sections in 1 mmol/l Tris–EDTA buffer (Tris-base: 648310, Sigma, USA; EDTA: E9884). The sections were then blocked with 5% bovine serum albumin (B2064; Sigma) and incubated overnight at 4 °C with an antibody targeting Kla (PTM-1401RM; PTM Biolabs, China). The following day, the sections were incubated with a horseradish peroxidase-labeled secondary antibody, namely, goat anti-rabbit IgG H&L (ab205718; Abcam, USA), at 37 °C for 30 min. After the try-488 tyrosine conversion reagent (Bry-try488; Runnerbio, China) was applied, a second round of heat-induced antigen retrieval and blocking was performed. Then, the sections were incubated with a primary antibody targeting histone H1 (18201-1-AP; ProteinTech, Wuhan, China), histone H2A (16441-1-AP; ProteinTech), histone H2B (15857-1-AP; ProteinTech), histone H3 (17168-1-AP; ProteinTech), or histone H4 (16047-1-AP; ProteinTech). These sections were then incubated with a labeled secondary antibody and the try-Cy3 tyrosine conversion reagent (Bry-Trycy3; Runnerbio), following the same procedure. Finally, the sections were mounted with 4′,6-diamidino-2-phenylindole-containing anti-fade mounting medium (P0131; Beyotime Biotechnology, China) and sealed. Fluorescence images were captured using a fluorescence microscope (Nikon Eclipse Ci-L, Nikon, Tokyo, Japan).

### RNA-sequencing and CUT&Tag

To systematically analyze the overall transcriptome map, RNA-sequencing (RNA-seq) was performed on the kidney tissues of the Con and DKD model mice. Total RNA was extracted using an RNeasy kit (Qiagen, Shanghai, China), and the concentration and purity of 1 μl of each RNA sample were determined with a NanoDrop8000 spectrophotometer (A260/A280 and A260/A230 ratios, respectively). RNA integrity was evaluated by an Agilent 2100 Bioanalyzer, and the RNA integrity number was used as the quality criterion. Single-stranded and double-stranded cDNA was synthesized from mRNA samples using SuperScript II (Invitrogen, CA, USA). High-quality total RNA (1 µg) was used as the starting material. A TruSeq RNA sample preparation kit was used for mRNA purification and fragmentation, first-strand cDNA synthesis, and second-strand cDNA synthesis. The double-stranded cDNA was then purified for end repair, dA tailing, adapter ligation, and DNA fragment enrichment. The size was checked using a DNA-specific chip such as an Agilent DNA-1000 on an Agilent Technologies 2100 Bioanalyzer. The libraries were quantified using Qubit (Invitrogen) according to the Qubit user guide. The libraries were then pooled and sequenced on a NovaSeq Xplus (Illumina) at a depth of ~20 M reads per sample.

For CUT&Tag, ~10^6^ cells were harvested and washed thoroughly to eliminate cellular debris and residual culture medium. Then, 100 μl of wash buffer was added to each sample to resuspend the cells. ConA beads were prewashed and resuspended in binding buffer. One hundred microliters of cells was added to activated ConA beads Pro and incubated at room temperature for 10 min. The cells were centrifuged briefly to collect the reaction solution, and the supernatant was discarded after the solution was clarified. Primary antibodies were added at the recommended dilution and incubated at 4 °C overnight. The sample was centrifuged briefly, and the supernatant was discarded after the solution was clarified. Afterward, secondary antibodies were added at a 1:100 dilution and incubated at room temperature for 30 min. Then, 200 μl of Dig-Wash Buffer was added, and the mixture was inverted several times to ensure sufficient mixing of the buffer with the cell–bead complex. This process was repeated twice (three times in total). Two microliters of PA-Tn5 transposomes was added to each sample and incubated at room temperature for 1 h. Then, 50 μl of diluted TTBL was added, and the samples were incubated at 37 °C for 1 h. The reactions were stopped by the addition of stop buffer (2 μl of 10% SDS, an appropriate amount of DNA spike-in, and proteinase K), and the samples were incubated at 55 °C for 10 min. Pretreated Pro DNA extraction beads were used for DNA extraction, including the binding, washing, and elution steps. The liberated DNA was subsequently purified, amplified by PCR, and subsequently further purified to generate high-fidelity, low-bias sequencing libraries. The final libraries were subsequently sequenced on the Illumina NovaSeq Xplus platform according to the manufacturer’s recommended workflow. The primary antibodies used in this study are listed in Supplementary Table 1. The data sets supporting this study are publicly available. The RNA-seq and CUT&Tag data have been deposited in the China National Center for Bioinformation (CNCB, https://www.cncb.ac.cn/) under the BioProject accession number PRJCA057026.

### Cell culture and intervention

Human glomerular endothelial cells (HGECs) and HK-2 cells were obtained from Procell (Wuhan, China) and cultured in DMEM (CM-H061; CM-0109; Procell) for serial subcultivation. Cells cultured in normal glucose (5 mM) for 6 days were designated the Con. Cells cultured in high-glucose (HG) medium (25 mM) for 6 days were designated the HG group. Glucose (5 mM) plus mannitol (20 mM) was used as an osmotic control (mannitol) to mitigate the effects of elevated osmotic pressure resulting from the HG treatment of cells. The lactate dehydrogenase A (LDHA) inhibitor OXA (HY-W013032A; MCE) was used in the cellular experiments. The cells in the OXA or HG + OXA group were incubated with 25 mM OXA for 3 days. Another LDHA inhibitor, STP (HY-103392; MCE), was used in the cellular experiments. Cells in the STP and HG + STP groups were incubated with 500 nM STP for 3 days. Cultured cells were treated with 10 mM lactate (HY-B2227; MCE) to further validate the involvement of lactate in HG-induced necroptosis. gRc (HY-N0042; MCE) was used to inhibit KRT18-induced H3K18la and H3K27la in hyperglycemic cells. The cells cultured under HG conditions were incubated with different concentrations of gRc (25, 50, 100, or 200 μM) for 3 days. The optimal concentration for the significant inhibitory effects of gRc on 25 mM glucose-induced H3K18la and H3K27la levels was determined.

### PI assays

A PI kit (A211-01; Vazyme Biotech, China) was used to label early-stage dead cells as green and late-stage cells as red according to the manufacturer’s instructions. Images were captured with a fluorescence microscope.

### Western blotting analysis

Tissue samples from the mouse model or cultured cells were homogenized using radioimmunoprecipitation assay lysis buffer (PC101; Epizyme Biotech, Shanghai, China) supplemented with phenylmethylsulfonyl fluoride (ST2573-5 g; Beyotime Biotechnology). Protein concentrations were measured using a BCA protein assay kit (P0009; Beyotime Biotechnology). Equal amounts of protein (20 μg) from each group were separated by SDS‒PAGE and subsequently transferred to polyvinylidene fluoride membranes (ISEQ00010; Millipore, Billerica, USA). The primary antibodies used in this study are listed in Supplementary Table 1.

### Short hairpin RNA and plasmid transfection

After the HGECs and HK-2 cells were seeded and reached 70‒80% confluence, they were transfected with plasmids or short hairpin RNA (shRNA) using Lipofectamine™ 3000 (L3000015; Thermo Fisher, USA). As specified in the user guide, 2,500 ng of plasmid or shRNA was initially mixed thoroughly with the P3000 Reagent. Next, this mixture was combined with Lipofectamine 3000. Finally, the resulting solution was added to the cell culture medium. The sequences of the shRNAs used in this study are shown in Supplementary Table 2.

### Quantitative real-time PCR (qPCR)

Total RNA was extracted from HGECs, HK-2 cells, or mouse tissues using a Universal RNA Purification Kit (EZB-RN4, EZbioscience, USA), and cDNA was prepared with HyperScript III RT SuperMix (R202-02; EnzyArtisan, Shanghai, China). Real-time PCR analysis was performed on an ABI7500 real-time PCR system (Applied Biosystems) using Universal SYBR qPCR Mix (EnzyArtisan). The transcript levels were normalized to those of β-actin. The primers used in this study are listed in Supplementary Table 3.

### In vitro lactylation reactions

In vitro lactylation reactions were performed by mixing purified GST-KRT18 protein (Ag1260; ProteinTech, Shanghai, China) with recombinant human His-histone H3 (Ag10644; ProteinTech) and lactate (5 mM; HY-B2227; MCE) in reaction buffer (50 mM HEPES, 25 mM KCl, 2 mM MgCl_2_, and 4 mM ATP, pH 7.5) for 3 h at 37 °C. Following the incubation, the samples were heated at 95 °C for 5 min to denature the proteins, which were subsequently analyzed by western blotting.

### GST pulldown assay

GST pulldown experiments were conducted using a GST pulldown kit (FI8804, FITGENE, Guangzhou, China). Specifically, the GST-KRT18 (Ag1260; ProteinTech) and His-histone H3 (Ag10644; ProteinTech) fusion proteins were used for the GST pulldown analysis. Equivalent quantities of these fusion proteins were co-incubated for 12 h at 4 °C in GST binding buffer (50 mM HEPES, pH 7.6; 50 mM NaCl; 0.1% Nonidet P-40; 5 mM EDTA; and 10% glycerol). Anti-GST beads were then combined with the fusion protein mixture and incubated for an additional 4 h. Following this step, the beads were washed three times with wash buffer (200 mM Tris–Cl, pH 8.0; 500 mM NaCl; 0.1 mM EDTA; 0.1% Triton X-100; and 0.4 mM phenylmethylsulfonyl fluoride), and the target proteins were detected via western blotting analysis.

### Co-immunoprecipitation assay

A conventional magnetic bead-based immunoprecipitation kit (88804; Thermo Scientific, USA) was used for co-immunoprecipitation (co-IP) experiments. HGECs and HK-2 cells were cultured in 100-mm dishes supplemented with 25 mM DMEM for 3 days. Afterward, the cells were harvested and lysed with cell lysis buffer (G2038; ServiceBio, Wuhan, China) supplemented with protease and phosphatase inhibitors (P1051; Beyotime Biotechnology) to prepare protein extracts. A volume of 30 μl of the supernatant was reserved as the input sample. The remaining supernatant was then incubated with the appropriate primary antibodies and Protein A/G Dynabeads at 4 °C overnight to immunoprecipitate endogenous proteins. Western blotting analysis was subsequently conducted to evaluate the input, IgG control, and IP samples.

### Isothermal titration calorimetry (ITC) assay

ITC experiments were performed using a MicroCal PEAQ-ITC calorimeter equipped with an automated system (Malvern) at 25 °C. For these measurements, purified KRT18 was placed in the ITC cell at a concentration of 5 µM, whereas 50 µM lactate or 50 µM gRc dissolved in 50 mM Tris–HCl and 150 mM NaCl was loaded into the syringe for automatic injection. Each titration consisted of an initial 1 µl injection followed by 19 consecutive injections of 2 µl each, with a 300-s interval between injections and stirring at 500 rpm. The resulting data were processed using ORIGIN data analysis software (MicroCal Software).

### Measurement of blood urea nitrogen (BUN) and serum creatinine (Scr), LDL-cholesterol, total cholesterol (TC), HDL-cholesterol, glycated albumin, lactate, and triglyceride (TG)

The levels of BUN were detected using a BUN assay kit (ADS-W-N013-96; AIDISHENG, China). The levels of Scr were measured using an Scr assay kit (ADS-W-FM034; AIDISHENG). The concentration of LDL-C in mouse serum was detected using an LDL-C assay kit (ADS-W-D012; AIDISHENG). The amount of TC in mouse serum was evaluated with a TC assay kit (ADS-W-ZF014; AIDISHENG). The levels of HDL-C in mouse serum were determined using an HDL-C assay kit (ADS-W-D011; AIDISHENG). Lactate concentrations in mouse serum were assessed using a lactate assay kit (ADS-W-T009-96; AIDISHENG). The concentration of TG in mouse serum was measured with a TG assay kit (ADS-W-ZF013; AIDISHENG).

### Pearson correlation analysis

Correlation analyses between BUN and H3K18la or H3K27la levels were conducted as follows. First, data normality was assessed using the Shapiro–Wilk test for all samples (*n* = 8) in the DKD group. Both BUN and histone lactylation measurements satisfied the assumption of normality (*P* > 0.05). Given the normally distributed data, Pearson correlation coefficients were calculated to quantify linear associations between BUN and each histone modification. Statistical significance was determined using two-tailed tests; a *P*-value <0.05 was considered statistically significant. Correlation results are reported as *r-*values with corresponding *P*-values. Scatter plots illustrating these correlations were generated using prime 9 (GraphPad Software, Inc.).

### Chromatin immunoprecipitation

Chromatin immunoprecipitation (ChIP) analysis was conducted using an EZ ChIP Kit (17-371; MERCK, USA). The cells were first rinsed with PBS and then treated with 1% formaldehyde at room temperature for 10 min to induce crosslinking between the DNA and proteins. The reaction was stopped by the addition of 2.5 M glycine. Chromatin was fragmented into appropriate sizes through sonication, which consisted of 10 cycles (each cycle included 7 s of sonication followed by a 7-s rest). The samples were divided and incubated overnight at 4 °C with antibodies against H3K18la, H3K27la, and KRT18 or with IgG as a negative control. On the next day, the immunoprecipitated complexes were collected using agarose beads and washed thoroughly, and the DNA‒protein crosslinks were reversed by incubation in a metal bath at 65 °C for 5‒6 h. Enriched DNA fragments were amplified by PCR using specific primers and analyzed by agarose gel electrophoresis. The sequences of the oligonucleotide primers are listed in Supplementary Table 4.

### Prediction of potential inhibitors of KRT18 and virtual screening

The 3D structure of human KRT18 (AlphaFold ID: AF-P05783-F1-v4) was obtained from the AlphaFold database (https://alphafold.ebi.ac.uk/entry/P05783). An analysis using the virtual screening workflow module revealed that the residues ASN322, SER323, and GLU326 serve as binding sites for lactate on KRT18. The receptor grid generation module was subsequently used to generate a grid file for KRT18, with the center positioned at the amino acid residues ASN322, SER323, and GLU326. The box dimensions were set to 20 Å × 20 Å × 20 Å. Finally, the HY-L022P FDA-Approved Drug Library Plus database (comprising 3,400 compounds in 2D format) was processed using the LigPrep module within Schrödinger software for tasks such as hydrogen addition and energy minimization. The resulting 3D structures were then generated for virtual screening purposes.

### Pathway enrichment analysis

The Kyoto Encyclopedia of Genes and Genomes (KEGG) database was used to detect enriched pathways, and the enrichment of differentially expressed genes was assessed using a two-tailed Fisher’s exact test. These pathways were then categorized into hierarchical classes on the basis of the information provided by the KEGG website.

### Statistical analysis

All the statistical analyses were performed using prime 9 (GraphPad Software, Inc.). The data are presented as the means ± SDs. The cell experiments were repeated at least five times, and the animal experiments were conducted with a minimum of eight animals per group to ensure representative results. Comparisons between two groups were conducted using an unpaired Student’s *t* test, whereas comparisons among multiple groups were performed using one-way analysis of variance followed by Tukey’s post hoc test. *P* < 0.05 was considered to indicate statistical significance.

## Results

### The levels of lactate, lysine lactylation (Kla), H3K18la, and H3K27la were increased in models and patients with DKD

**Fig. 1:**
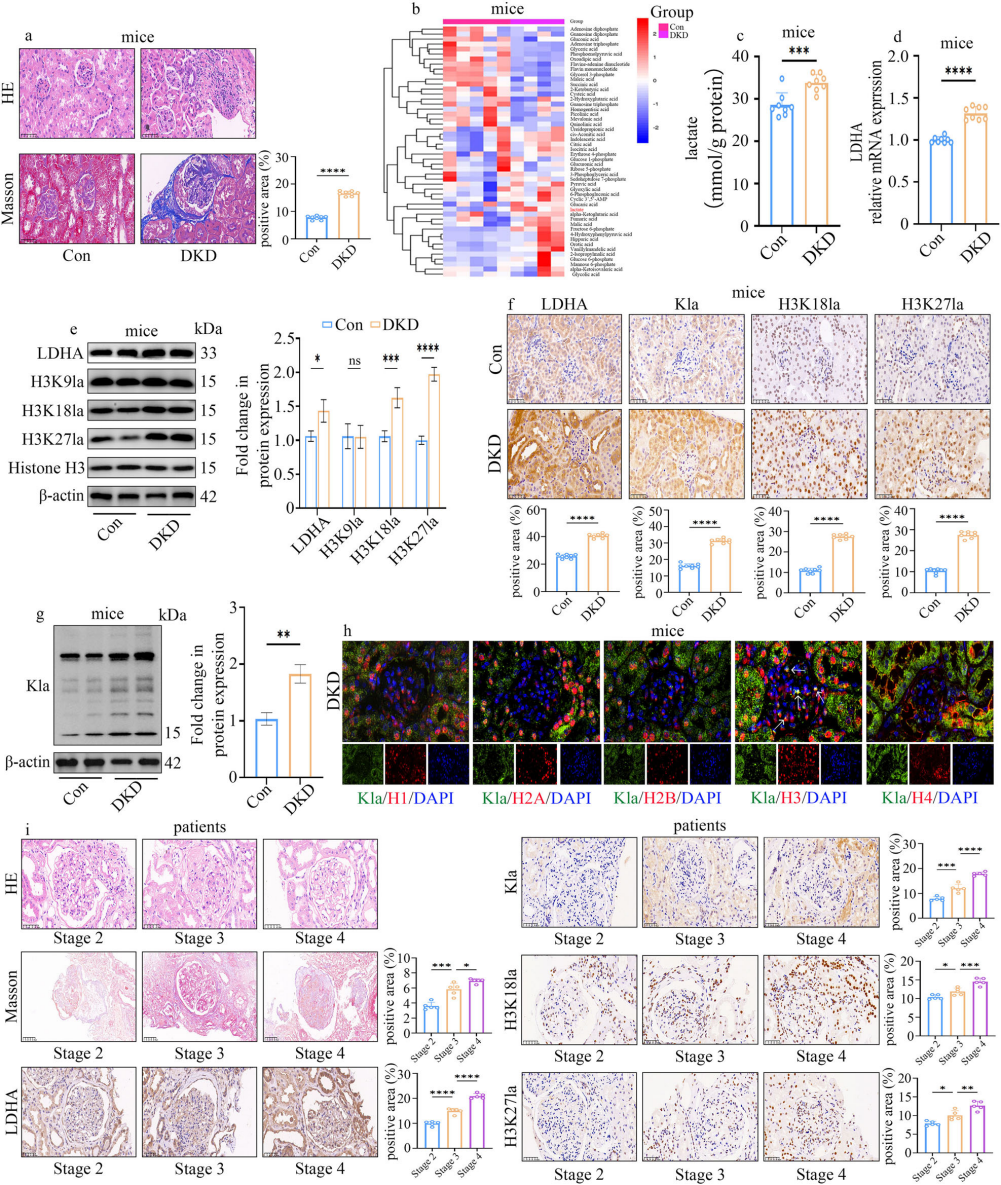
The levels of lactate, lysine lactylation (Kla), H3K18la, and H3K27la are increased in models and patients with diabetic kidney disease. **a** Representative microscopy images showing hematoxylin and eosin (HE) and Masson’s trichrome staining of kidney biopsy samples from control (Con) and diabetic kidney disease (DKD) model mice (scale bar, 50 μm). HE staining of renal tissue from DKD model mice revealed marked glomerular enlargement and adaptive tubular hypertrophy. Moreover, Masson’s trichrome staining revealed greater fibrosis in the renal tissues of DKD model mice than in those of control mice (data are presented as the mean ± SD; *n* = 8 per group). **b** The levels of kidney metabolites in DKD model mice were normalized to those in control mice and are shown in heatmaps, with the color scale indicating relative differences (*n* = 5 in the Con group and *n* = 4 in the DKD group). **c** Lactate levels were increased in DKD model mice compared with control mice (data are presented as the mean ± SD; *n* = 8 per group). **d** Quantitative PCR assays indicated that the mRNA levels of lactate dehydrogenase A (LDHA) were increased in the kidneys of DKD model mice (data are presented as the mean ± SD; *n* = 8 per group). **e** Western blotting revealed that the protein levels of H3K18la, H3K27la, and LDHA were increased in the kidneys of DKD model mice; however, H3K9la levels did not change significantly. **f** Representative images of immunohistochemistry (IHC) staining for LDHA, Kla, H3K18la, and H3K27la in renal biopsy samples from control group and DKD model mice (scale bar, 50 μm). The levels of LDHA, Kla, H3K18la, and H3K27la were increased in the kidneys of DKD model mice (data are presented as the mean ± SD; *n* = 8 per group). **g** Western blotting revealed that the protein levels of Kla were increased in the kidneys of DKD model mice. **h** Double immunofluorescence staining for Kla and histones H1 (H1), H2A, H2B, H3, or H4 verified the strong colocalization of Kla with H3 in the kidneys of DKD model mice, which indicated that histone lactylation modifications predominantly occur on H3 in DKD. **i** Representative images of HE staining, Masson’s trichrome staining, and IHC staining for LDHA, Kla, H3K18la, and H3K27la in renal biopsy specimens from patients with DKD in the present study (scale bar, 50 μm). HE staining indicated that glomerular sclerosis intensified progressively and that tubulointerstitial lesions transitioned from multifocal to diffuse involvement as the DKD stage progressed. Masson’s trichrome staining revealed an increase in collagen deposition and interstitial fibrosis as the DKD stage progressed. IHC data indicated that LDHA, Kla, H3K18la, and H3K27la levels in renal biopsy samples from patients with DKD gradually increased with increasing DKD stage (data are presented as the mean ± SD; *n* = 5 per group). DAPI, 4′,6-diamidino-2-phenylindole; ns, not significant. *P < 0.05 and **P < 0.01.

We initially developed a DKD mouse model to explore the mechanisms underlying DKD. Serum biochemical analysis revealed significantly elevated blood glucose, BUN, and Scr levels in DKD model mice (Supplementary Table 5). In addition, compared with control mice, DKD model mice displayed signs of renal damage and fibrosis (Fig. 1a). Various glucose metabolic products have essential roles in the development of diabetes-related complications. Hence, a metabolomic analysis was conducted in this investigation. Metabolomic analysis revealed increased lactate levels in the kidney tissues of DKD model mice (Fig. 1b and Supplementary Table 6). Moreover, direct lactate measurement in renal tissue corroborated the trends observed in the metabolomic data (Fig. 1c). Previous studies have highlighted lactate as a significant contributor to diabetes-associated complications^12^. Additionally, LDHA catalyzes the conversion of pyruvate to lactate through the glucose metabolic pathway. Accordingly, we examined LDHA expression in the kidneys of DKD model mice and detected increased LDHA levels in these tissues (Fig. 1d–f). Previous studies have shown that lactate-induced Kla and histone lactylation are crucial in the pathogenesis of DKD^19,20^. Consistent with these reports, our findings revealed increased lactate-mediated Kla levels in the kidneys of DKD model mice (Fig. 1f, g). Double immunofluorescence staining was performed for Kla and histones H1, H2A, H2B, H3, and H4 to further identify the specific histone lactylation modifications involved in DKD. Our results revealed strong colocalization of Kla with histone H3 in the kidneys of DKD model mice (Fig. 1h). These observations suggest that histone lactylation modifications primarily occur on H3 in DKD. Next, we evaluated H3K9la, H3K18la, and H3K27la levels in DKD model mice. Western blotting and IHC analyses revealed significant increases in H3K18la and H3K27la levels in the kidneys of DKD model mice, whereas H3K9la expression remained stable (Fig. 1e, f). In addition, the expression of H3K18la and H3K27la was positively correlated with BUN levels in DKD model mice (Supplementary Fig. 1a,b).

Next, we developed DKD cellular models utilizing HGECs and HK-2 cells. HG concentrations increased lactate accumulation; LDHA, Kla, H3K18la, and H3K27la levels; and cell death in hyperglycemic cells (Supplementary Fig. 1c–l). However, the level of H3K9la remained largely unchanged (Supplementary Fig. 1e,j).

The features of the patients with DKD included in this study are outlined in Supplementary Table 7. These patients were classified as having stage 2, 3, or 4 disease according to their eGFR. Images of HE-stained samples from patients with DKD are shown in Fig. 1i. Masson’s trichrome staining revealed an increase in collagen deposition and interstitial fibrosis as the DKD stage progressed (Fig. 1i). In agreement with these observations, the levels of LDHA, Kla, H3K18la, and H3K27la in renal biopsy samples from patients with DKD increased gradually with increasing DKD stage (Fig. 1i).

### Increases in H3K18la and H3K27la levels mediated by lactate accumulation are involved in cell death and renal dysfunction in models with DKD

**Fig. 2:**
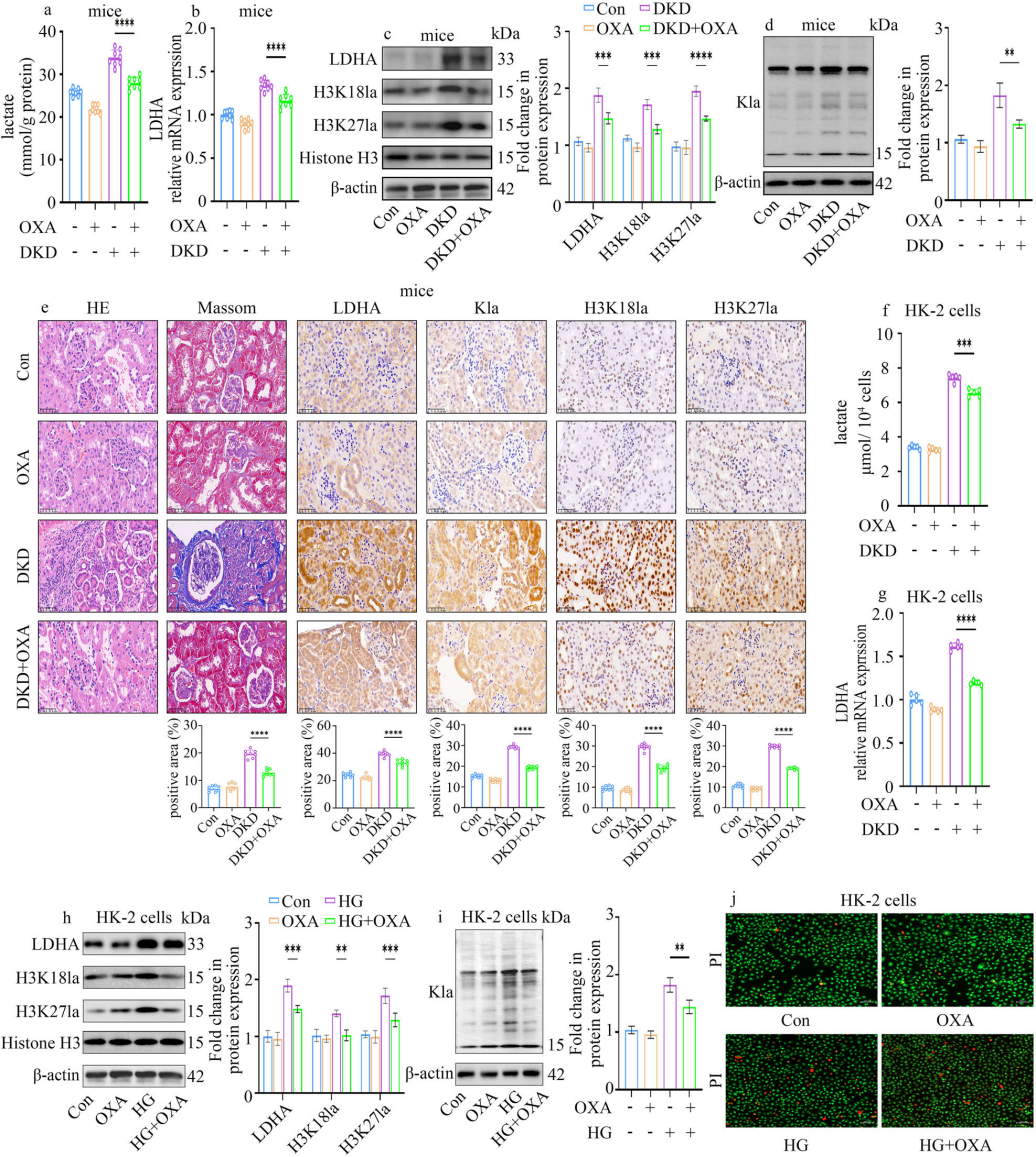
The suppression of lactate accumulation and reduction in H3K18la and H3K27la levels alleviates cell death and renal dysfunction in models with diabetic kidney disease. **a** Oxamate (OXA) treatment decreased lactate accumulation in the renal tissues of diabetic kidney disease (DKD) model mice (data are presented as the mean ± SD; *n* = 8 per group). **b** OXA treatment decreased lactate dehydrogenase A (LDHA) mRNA levels in the renal tissues of DKD model mice (data are presented as the mean ± SD; *n* = 8 per group). **c** OXA treatment decreased LDHA, H3K18la, and H3K27la levels in the renal tissues of DKD model mice. **d** OXA treatment reduced lysine lactylation (Kla) levels in the renal tissues of DKD model mice. **e** Representative images of hematoxylin and eosin (HE) staining, Masson’s trichrome staining and immunohistochemistry staining for LDHA, Kla, H3K18la, and H3K27la in the renal tissues of control (Con), oxamate (OXA), DKD, and DKD + OXA model mice (scale bar, 50 μm). OXA treatment attenuated renal injury and fibrosis and decreased LDHA, Kla, H3K18la, and H3K27la levels in the renal tissues of DKD model mice (data are presented as the mean ± SD; *n* = 8 per group). **f** OXA treatment decreased lactate accumulation in hyperglycemic HK-2 cells (data are presented as the mean ± SD; *n* = 5 per group). **g** OXA treatment decreased LDHA mRNA levels in hyperglycemic HK-2 cells (data are presented as the mean ± SD; *n* = 5 per group). **h** OXA treatment decreased LDHA, H3K18la, and H3K27la levels in hyperglycemic HK-2 cells. **i** OXA treatment reduced Kla levels in hyperglycemic HK-2 cells. **j** OXA treatment inhibited the death of hyperglycemic HK-2 cells. HG, high glucose. *P < 0.05 and **P < 0.01.

We confirmed that lactate-induced H3K18la and H3K27la expression was involved in DKD by treating DKD model mice with the LDHA inhibitor OXA or STP to reduce lactate synthesis. Our results indicated that OXA or STP treatment decreased lactate synthesis in renal tissues (Fig. 2a and Supplementary Fig. 2a); decreased LDHA, Kla, H3K18la, and H3K27la levels (Fig. 2b–e and Supplementary Fig. 2b–e); attenuated renal injury and fibrosis (Fig. 2e and Supplementary Fig. 2e); and reduced serum BUN and Scr levels (Supplementary Table 5) in DKD model mice. Consistently, OXA or STP treatment decreased lactate levels; decreased LDHA, Kla, H3K18la, and H3K27la levels; and inhibited cell death in hyperglycemic cells (Fig. 2f–j and Supplementary Figs. 3 and 4).

Next, we treated DKD model mice with lactate. Our data indicated that lactate administration to DKD model mice led to increased lactate levels in renal tissues (Supplementary Fig. 5a); upregulated Kla, H3K18la, and H3K27la levels; exacerbated renal injury and fibrosis; and further increased serum BUN and Scr levels in the DKD model (Supplementary Fig. 5b–d and Supplementary Table 5). Similarly, lactate treatment upregulated lactate levels; increased Kla, H3K18la, and H3K27la levels; and increased cell death in hyperglycemic cells (Supplementary Fig. 6).

We subsequently constructed lentiviral plasmids expressing wild-type H3 and a H3 mutation in which K18/27 was replaced with arginine (H3-K18/27R) to further verify the involvement of lactate-mediated H3K18la and H3K27la in HG-mediated cell death. Notably, the effects of HG on increasing H3K18la and H3K27la levels, as well as cell death, were significantly attenuated or abolished after the overexpression of the H3-K18/27R mutation in cells (Supplementary Fig. 7a–d). These findings indicate that lactate-induced H3K18la and H3K27la have critical roles in renal injury in DKD models.

Furthermore, we began to explore the mechanism by which LDHA inhibitors suppress LDHA transcription. Given the significant role of lactate-mediated H3K18la and H3K27la in gene transcriptional regulation, CUT&Tag data from HGECs exposed to HG conditions were analyzed (Supplementary Tables 8 and 9). The results revealed that H3K18la and H3K27la were enriched in the LDHA promoter region (Supplementary Fig. 7e), a finding confirmed by ChIP experiments (Supplementary Fig. 7f). These results suggest that inhibiting lactate production reduces H3K18/27la levels, thereby suppressing LDHA transcription.

### Lactate-mediated H3K18la and H3K27la participate in DKD by triggering necroptosis

**Fig. 3:**
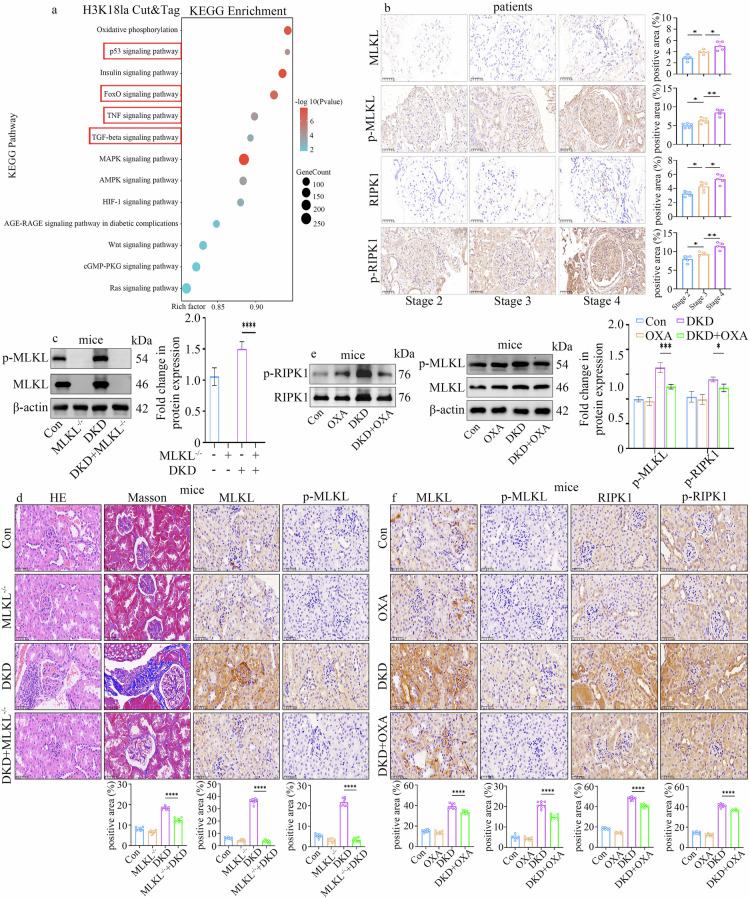
Lactate-mediated H3K18la and H3K27la participate in diabetic kidney disease by triggering necroptosis. **a** Analysis of the molecular function of H3K18la using CUT&Tag data and the Kyoto Encyclopedia of Genes and Genomes (KEGG) database. **b** Immunohistochemistry (IHC) staining revealed a progressive increase in the levels of p-RIPK1 and p-MLKL with increasing diabetic kidney disease (DKD) stage (data are presented as the mean ± SD; *n* = 5 per group). **c** Western blotting was performed on the kidneys of control (Con), MLKL^−/−^, DKD, and DKD + MLKL^−/−^ model mice. **d** Representative images of hematoxylin and eosin (HE) staining, Masson’s trichrome staining and IHC staining for MLKL and p-MLKL in renal biopsy samples from control (Con), MLKL^−/−^, DKD, and DKD + MLKL^−/−^ model mice (scale bar, 50 μm). Compared with DKD model mice, DKD + MLKL^−/−^ model mice presented less renal injury and fibrosis (data are presented as the mean ± SD; *n* = 8 per group). **e** Western blotting indicated that oxamate (OXA) treatment decreased p-MLKL and p-RIPK1 levels in renal biopsy samples from DKD model mice. **f** Representative images of IHC staining for MLKL, p-MLKL, RIPK1, and p-RIPK1 in renal biopsy samples from Con, OXA, DKD, and DKD + OXA model mice (scale bar, 50 μm). Compared with DKD model mice, DKD + OXA model mice presented lower p-MLKL and p-RIPK1 levels (data are presented as the mean ± SD; *n* = 8 per group). TGF-β transforming growth factor-β, TNF tumor necrosis factor, MAPK mitogen-activated protein kinase, AMPK AMP-activated protein kinase, HIF-1 hypoxia-inducible factor 1, AGE-RAGE advanced glycation end products-receptor for advanced glycation end products, cGMP-PKG cyclic guanosine monophosphate-protein kinase G. *P < 0.05 and **P < 0.01.

Considering the aforementioned findings that H3K18la and H3K27la are critical for cell death in DKD, we further analyzed CUT&Tag data (Supplementary Tables 8 and 9). An analysis of H3K18la and H3K27la CUT&Tag data revealed that KEGG pathways related to the tumor necrosis factor, FoxO, transforming growth factor-β, and p53 signaling pathways were enriched (Fig. 3a, Supplementary Fig. 8a and Supplementary Tables 10 and 11). Notably, these pathways have crucial roles in cell death, including apoptosis and necroptosis^33–35^. To further determine which type of cell death has a crucial role in DKD, relevant indicators were examined. Our data indicated that in hyperglycemic cells, the levels of p-MLKL and p-RIPK1 increased, whereas the expression of BAX, bcl-2, and caspase 3 did not change significantly (Supplementary Fig. 8b). These data indicate that necroptosis has a crucial role in DKD. Furthermore, the effects of HG on increasing p-MLKL and p-RIPK1 levels were significantly attenuated or abolished after the overexpression of the H3-K18/27R mutant in cells (Supplementary Fig. 8c). These data indicate that lactate-mediated H3K18la and H3K27la have crucial roles in necroptosis in hyperglycemic cells.

We subsequently analyzed necroptosis-related markers in renal tissue sections derived from patients with DKD. The results demonstrated a progressive increase in p-RIPK1 and p-MLKL levels as the DKD stage advanced (Fig. 3b). We utilized MLKL-deficient mice to establish a DKD model (DKD + MLKL^−/−^) and verify the critical role of necroptosis in DKD (Fig. 3c). Compared with conventional DKD model mice, DKD + MLKL^−/−^ mice exhibited attenuated renal injury (Fig. 3d), reduced fibrosis (Fig. 3d), and reduced serum BUN and Scr levels (Supplementary Table 5). These data suggest that necroptosis significantly contributes to the pathogenesis of DKD.

We further explored whether HG-induced H3K18la and H3K27la expression is involved in DKD via the induction of necroptosis by evaluating the effects of OXA or STP on necroptosis in the kidneys of DKD model mice and hyperglycemic cells. Our results indicated that OXA or STP treatment resulted in reduced levels of p-MLKL and p-RIPK1 in both the kidneys of DKD model mice and hyperglycemic cells (Fig. 3e, f and Supplementary Figs. 8d and 9). Moreover, lactate treatment resulted in increased p-MLKL and p-RIPK1 levels in DKD models (Supplementary Fig. 10). These data suggest that lactate-induced H3K18la and H3K27la have critical roles in the progression of DKD by triggering necroptosis.

### Lactate-mediated H3K18la and H3K27la expression modulates Fas transcription to trigger necroptosis in DKD

**Fig. 4:**
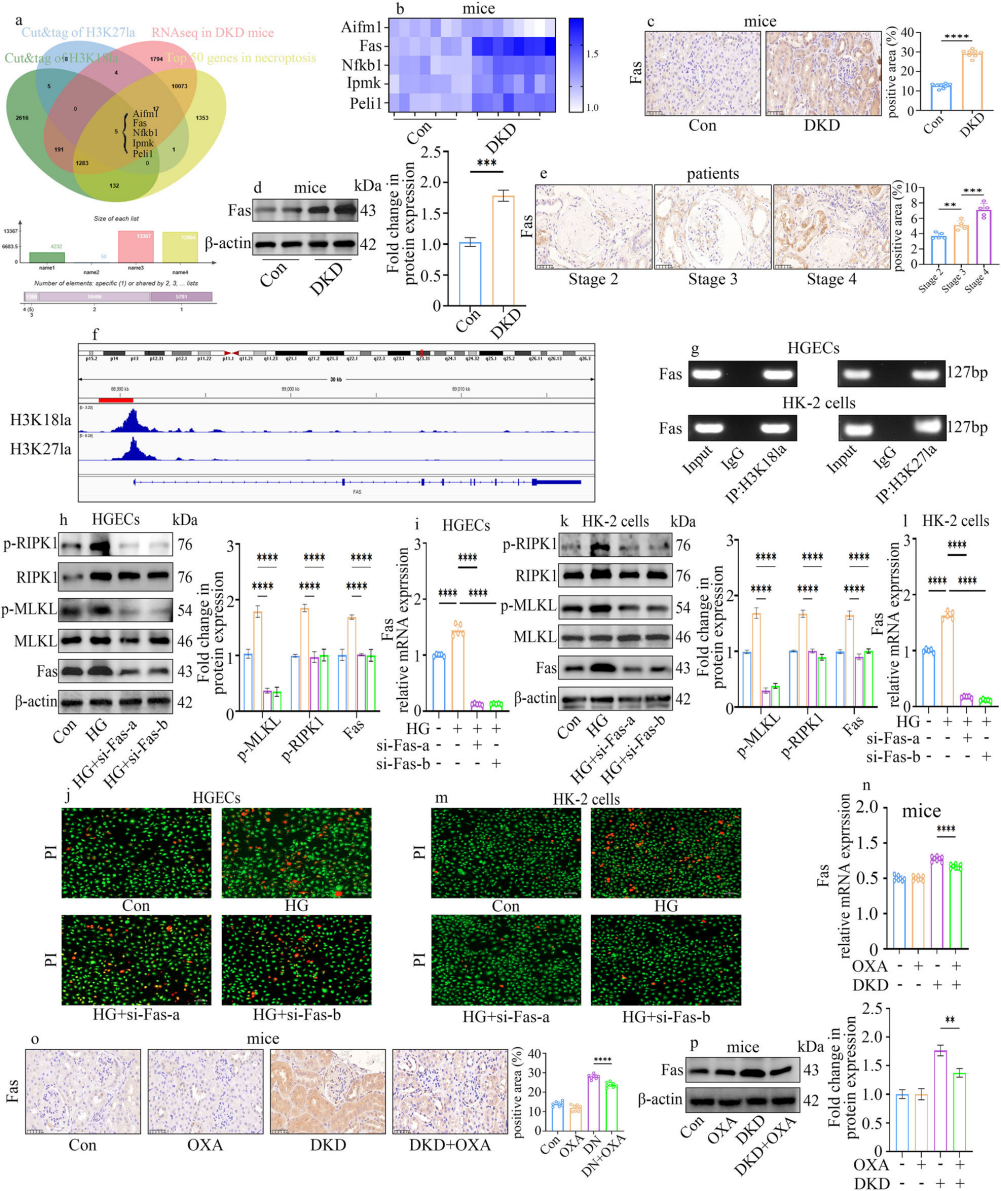
Lactate-mediated H3K18la and H3K27la expression modulates Fas transcription to trigger necroptosis in diabetic kidney disease. **a** Venn diagram of the RNA-sequencing data for genes in the kidneys of diabetic kidney disease (DKD) model mice and H3K18la and H3K27la CUT&Tag data, as well as the top 50 necroptosis-related genes obtained from GeneCards. **b** Quantitative PCR (qPCR) assays were performed on the kidneys of DKD model mice (data are presented as the mean ± SD; *n* = 8 per group). **c** Representative images of immunohistochemistry (IHC) staining for Fas in renal biopsy samples from control (Con) and DKD model mice (scale bar, 50 μm). Fas levels were increased in the kidneys of DKD model mice (data are presented as the mean ± SD; *n* = 8 per group). **d** Western blotting indicated that Fas protein levels were increased in the kidneys of DKD model mice. **e** IHC staining revealed a progressive increase in the level of Fas with increasing DKD stage (scale bar, 50 μm; data are presented as the mean ± SD; *n* = 5 per group). **f** CUT&Tag data indicating that H3K18la and H3K27la may occupy the promoter region of Fas. **g** Chromatin immunoprecipitation assays verified that H3K18la and H3K27la occupied the promoter region of Fas in cells. **h** Western blotting revealed that Fas silencing inhibited necroptosis in hyperglycemic HGECs. **i** qPCR was performed on hyperglycemic HGECs (data are presented as the mean ± SD; *n* = 5 per group). **j** PI staining indicated that Fas silencing inhibited the death of hyperglycemic HGECs. **k** Western blotting revealed that Fas silencing inhibited necroptosis in hyperglycemic HK-2 cells. **l** qPCR was performed on hyperglycemic HK-2 cells (data are presented as the mean ± SD; *n* = 5 per group). **m** PI staining indicated that Fas silencing inhibited the death of hyperglycemic HK-2 cells. **n** qPCR indicated that oxamate (OXA) treatment reduced Fas mRNA levels in the kidneys of DKD model mice (data are presented as the mean ± SD; *n* = 8 per group). **o** IHC staining indicated that OXA treatment reduced Fas levels in the kidneys of DKD model mice (data are presented as the mean ± SD; *n* = 8 per group). **p** Western blotting indicated that OXA treatment reduced Fas levels in the kidneys of DKD model mice (data are presented as the mean ± SD; *n* = 8 per group). HG, high glucose. *P < 0.05 and **P < 0.01.

We analyzed RNA-seq data from the kidneys of DKD model mice (Supplementary Table 12), CUT&Tag data for H3K18la and H3K27la (Supplementary Tables 8 and 9), and the top 50 necroptosis-related genes (Supplementary Table 13) obtained from GeneCards (https://www.genecards.org/) to elucidate the precise mechanism by which lactate-mediated H3K18la and H3K27la contribute to necroptosis in DKD. Our data indicated that Fas, apoptosis-inducing factor 1 (Aifm1), nuclear factor kappa B subunit 1 (Nfkb1), inositol polyphosphate multikinase (Ipmk), and Pellino1 (Peli1) may contribute to necroptosis in DKD (Fig. 4a). Among these five genes, Fas mRNA expression was most significantly increased in the kidneys of DKD model mice (Fig. 4b). Moreover, Fas levels were increased in DKD model mice (Fig. 4c, d) and hyperglycemic cells (Supplementary Fig. 11a–d). Consistent with these findings, Fas expression gradually increased in the kidneys of patients with DKD as the DKD stage advanced (Fig. 4e). CUT&Tag data indicated that H3K18la and H3K27la may occupy the promoter region of Fas (Fig. 4f and Supplementary Tables 8 and 9), a finding that was confirmed by a ChIP assay (Fig. 4g). Furthermore, Fas silencing attenuated necroptosis in hyperglycemic cells (Fig. 4h–m). Moreover, OXA or STP treatment decreased Fas levels in the kidneys of DKD model mice and hyperglycemic cells (Fig. 4n–p and Supplementary Figs. 11e–h and 12a–g). Consistently, lactate treatment increased Fas levels in the kidneys of DKD model mice (Supplementary Fig. 12h–j) and hyperglycemic cells (Supplementary Fig. 12k–n). In addition, the ability of HG levels to increase Fas expression was significantly attenuated or abolished after the H3-K18/27R mutant was overexpressed in cells (Supplementary Fig. 13). These data indicate that lactate-mediated H3K18la and H3K27la level modulates Fas transcription to trigger necroptosis in DKD.

### KRT18 acts as a lactyltransferase that lactylates H3K18 and H3K27 in DKD

**Fig. 5:**
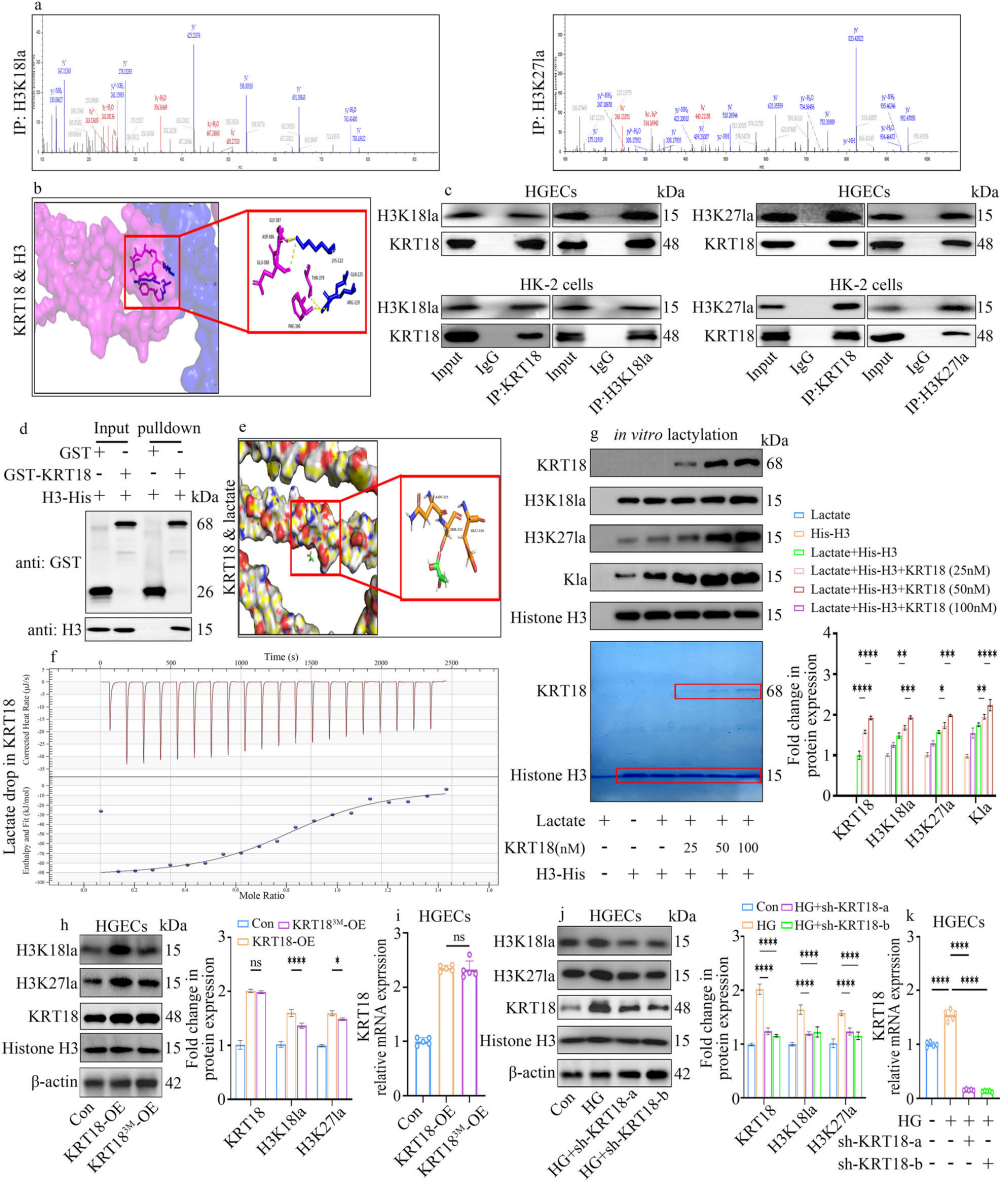
KRT18 acts as a lactyltransferase that lactylates H3K18 and H3K27 in diabetic kidney disease. **a** Co-immunoprecipitation (co-IP) coupled with mass spectrometry analysis was conducted. Our data indicated that KRT18 may be associated with H3K18la and H3K27la. **b** Molecular docking experiments suggested that KRT18 can directly bind to H3. **c** The interaction between KRT18 and H3K18la or H3K27la in cells was verified by co-IP. **d** A GST pulldown assay indicated that KRT18 directly binds to H3. **e** Molecular docking analysis indicated that KRT18 directly binds to lactate. **f** Isothermal titration calorimetry verified that KRT18 directly binds to lactate. **g** An in vitro lactylation assay indicated that KRT18 uses lactate as a substrate to lactylate H3K18 and H3K27 in a concentration-dependent manner. **h** Western blotting revealed that KRT18 overexpression increased H3K18la and H3K27la levels in cells. However, the KRT18^3M^ mutant abolished the KRT18-induced increase in H3K18la and H3K27la levels. **i** Quantitative PCR assays were performed to measure the KRT18 mRNA levels in cells subjected to the corresponding treatments (data are presented as the mean ± SD; *n* = 5 per group). **j** Western blotting revealed that KRT18 silencing decreased H3K18la and H3K27la levels in cells exposed to high glucose concentrations. **k** qPCR assays were performed to measure KRT18 mRNA expression levels in cells subjected to the corresponding treatments (data are presented as the mean ± SD; *n* = 5 per group). Con, control; ns, not significant. *P < 0.05 and **P < 0.01.

The H3K18la and H3K27la modifications are regulated by specific enzymes that add or remove these marks. Therefore, identifying the enzymes that regulate H3K18la and H3K27la is critical for further exploration of their roles in the pathogenesis of DKD. We conducted co-IP coupled with mass spectrometry analysis to identify potential enzymes involved in modulating H3K18la and H3K27la. Our findings suggested that members of the KRT family, including KRT18 and KRT5, may interact with H3K18la and H3K27la (Fig. 5a, Supplementary Fig. 14a and Supplementary Tables 14 and 15). Consistent with these findings, crystallographic analyses revealed that KRT18 directly interacts with H3 (Fig. 5b), a finding supported by the results of co-IP and GST pulldown experiments (Fig. 5c,d). Additionally, molecular docking studies demonstrated that KRT18 binds to lactate (Fig. 5e), which was further validated using ITC (Fig. 5f). An in vitro lactylation assay revealed that KRT18 utilizes lactate as a substrate to modify H3K18 and H3K27 through lactylation in a concentration-dependent manner (Fig. 5g). Similarly, in vitro lactylation assays revealed that KRT5 also mediated H3K18la and H3K27la (Supplementary Fig. 14b). These data indicate that members of the KRT family may act as lactyltransferases that participate in DKD.

We subsequently evaluated the expression of KRT18 and KRT5 in DKD model mice. Our data indicated that KRT18 expression was increased in the kidneys of DKD model mice (Supplementary Fig. 15a–c), whereas KRT5 expression did not change (Supplementary Fig. 15a). In addition, KRT18 expression increased in hyperglycemic cells (Supplementary Fig. 15d–g). Similarly, the levels of KRT18 in renal biopsy samples from patients with DKD increased gradually with increasing DKD stage (Supplementary Fig. 15h). On the basis of these observations, this study focussed primarily on investigating the function of KRT18 in DKD.

Next, both loss-of-function and gain-of-function approaches were used to confirm the role of KRT18 as a lactyltransferase that modulates H3K18la and H3K27la ex vivo. Our results indicated that KRT18 overexpression increased the levels of H3K18la and H3K27la in cells (Fig. 5h, i and Supplementary Fig. 16a,b). Crystallographic data revealed ASN322, SER323, and GLU326 as key lactate-binding residues within KRT18 (Fig. 5e). On the basis of this information, we hypothesized that these residues are essential for the lactyltransferase activity of KRT18. A mutant plasmid (KRT18^3M^, N322R, S323R, and Q326G) targeting these three sites was constructed. KRT18^3M^ did not affect the levels of H3K18la or H3K27la (Fig. 5h, i and Supplementary Fig. 16a,b), underscoring the importance of lactate binding for KRT18-mediated lactylation. Furthermore, silencing KRT18 reduced H3K18la and H3K27la levels in hyperglycemic cells (Fig. 5j, k and Supplementary Fig. 16c,d). Collectively, these findings suggest that KRT18 functions as a lactyltransferase and uses lactate as a substrate to directly lactylate H3K18 and H3K27.

### KRT18 acts as a lactyltransferase to trigger necroptosis in DKD by modulating Fas transcription

Next, we determined whether KRT18-induced H3K18la and H3K27la participate in necroptosis in DKD by modulating Fas transcription. Our data revealed that KRT18 silencing decreased Fas levels and inhibited necroptosis in hyperglycemic cells (Supplementary Fig. 17a–f). In addition, KRT18 overexpression increased Fas expression and promoted necroptosis in cells (Supplementary Fig. 17g–n). Moreover, the effect of KRT18 overexpression on necroptosis was abrogated by Fas silencing (Supplementary Fig. 17g–n). Furthermore, KRT18^3M^ did not affect Fas transcription or necroptosis in cells (Supplementary Fig. 18a–f). ChIP assays also confirmed that KRT18 was enriched at the promoter region of Fas (Supplementary Fig. 18g). These data indicate that KRT18 lactylates H3K18 and H3K27 to activate Fas transcription, thus triggering necroptosis in hyperglycemic cells.

**Fig. 6:**
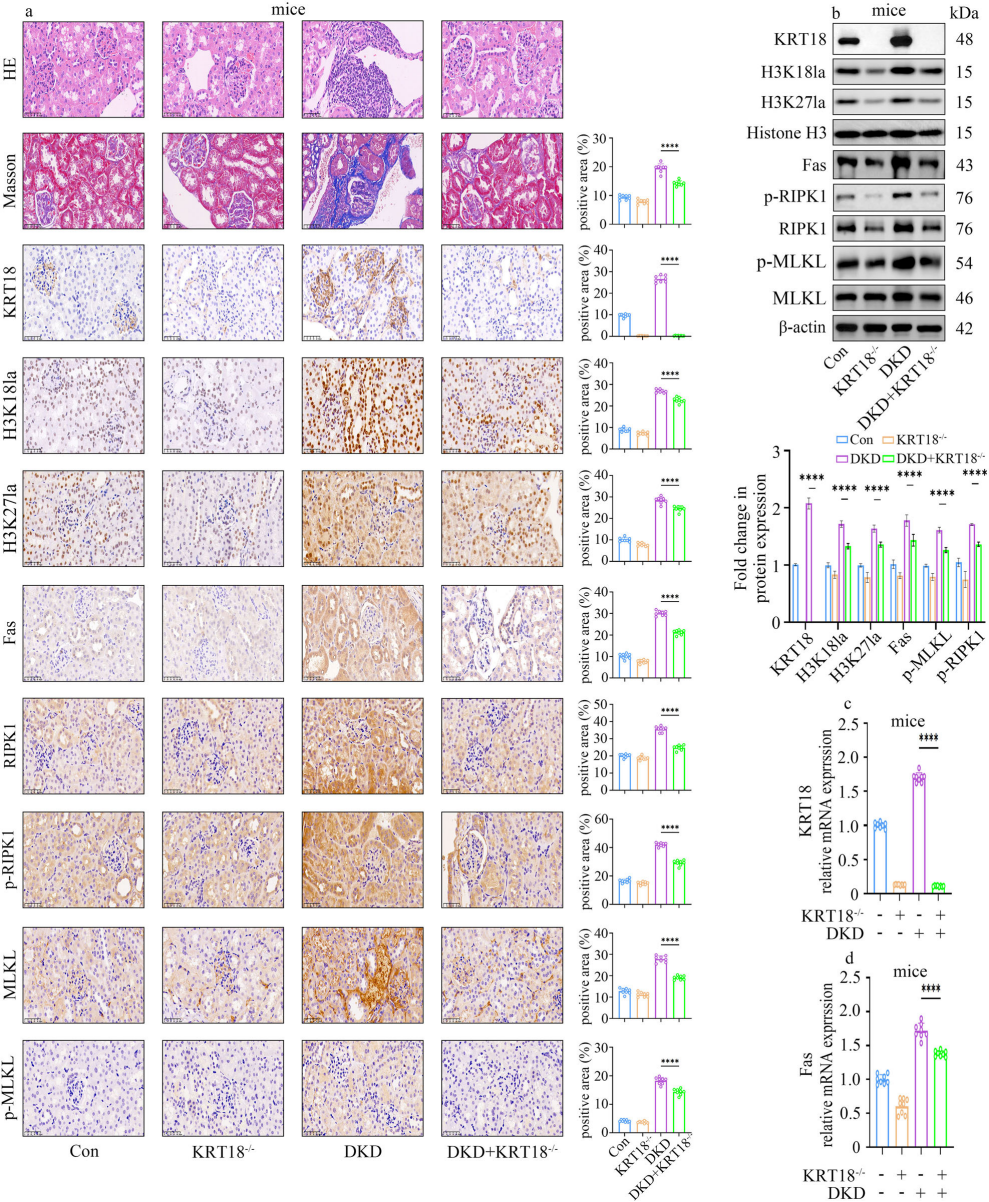
KRT18 suppression decreases H3K18la, H3K27la, and Fas expression and inhibits necroptosis in diabetic kidney disease model mice. **a** Representative images of hematoxylin and eosin (HE) staining, Masson’s trichrome staining, and immunohistochemistry staining for KRT18, H3K18la, H3K27la, Fas, p-RIPK1, RIPK1, p-MLKL, and MLKL in renal biopsy samples from control (Con), KRT18^−/−^, diabetic kidney disease (DKD), and DKD + KRT18^−/−^ model mice (scale bar, 50 μm). The levels of KRT18, H3K18la, H3K27la, Fas, p-RIPK1, RIPK1, p-MLKL, and MLKL were increased in the kidneys of DKD model mice. Moreover, the suppression of KRT18 expression reduced H3K18la, H3K27la, and Fas levels; mitigated necroptosis; and attenuated renal injury (data are presented as the mean ± SD; *n* = 8 per group). **b** Western blotting revealed that the protein levels of KRT18, H3K18la, H3K27la, Fas, p-RIPK1, RIPK1, p-MLKL, and MLKL were increased in the kidneys of DKD model mice. Moreover, the suppression of KRT18 expression resulted in reduced H3K18la, H3K27la, and Fas levels and mitigated necroptosis in DKD model mice. **c** KRT18 mRNA expression in the kidneys of the mice in this study was detected via a quantitative PCR assay (data are presented as the mean ± SD; *n* = 8 per group). **d** Quantitative PCR assays revealed that the Fas mRNA level was increased in the kidneys of DKD model mice, but this effect was reversed by KRT18 suppression (data are presented as the mean ± SD; *n* = 8 per group). *P < 0.05 and **P < 0.01.

Furthermore, we conducted experiments using KRT18^−/−^ mice to confirm that KRT18 modulates H3K18la and H3K27la to trigger necroptosis through the activation of Fas transcription in vivo. Our findings revealed that the suppression of KRT18 expression resulted in reduced H3K18la, H3K27la, and Fas levels (Fig. 6a–d), mitigated necroptosis and renal injury (Fig. 6a, b), and reduced serum BUN and Scr levels in DKD model mice (Supplementary Table 5). These observations suggest that KRT18 stimulates Fas transcription by lactylating H3K18 and H3K27, thereby triggering necroptosis in DKD.

### gRc competitively antagonized KRT18-induced H3K18la and H3K27la expression to inhibit Fas transcription and necroptosis in DKD

**Fig. 7:**
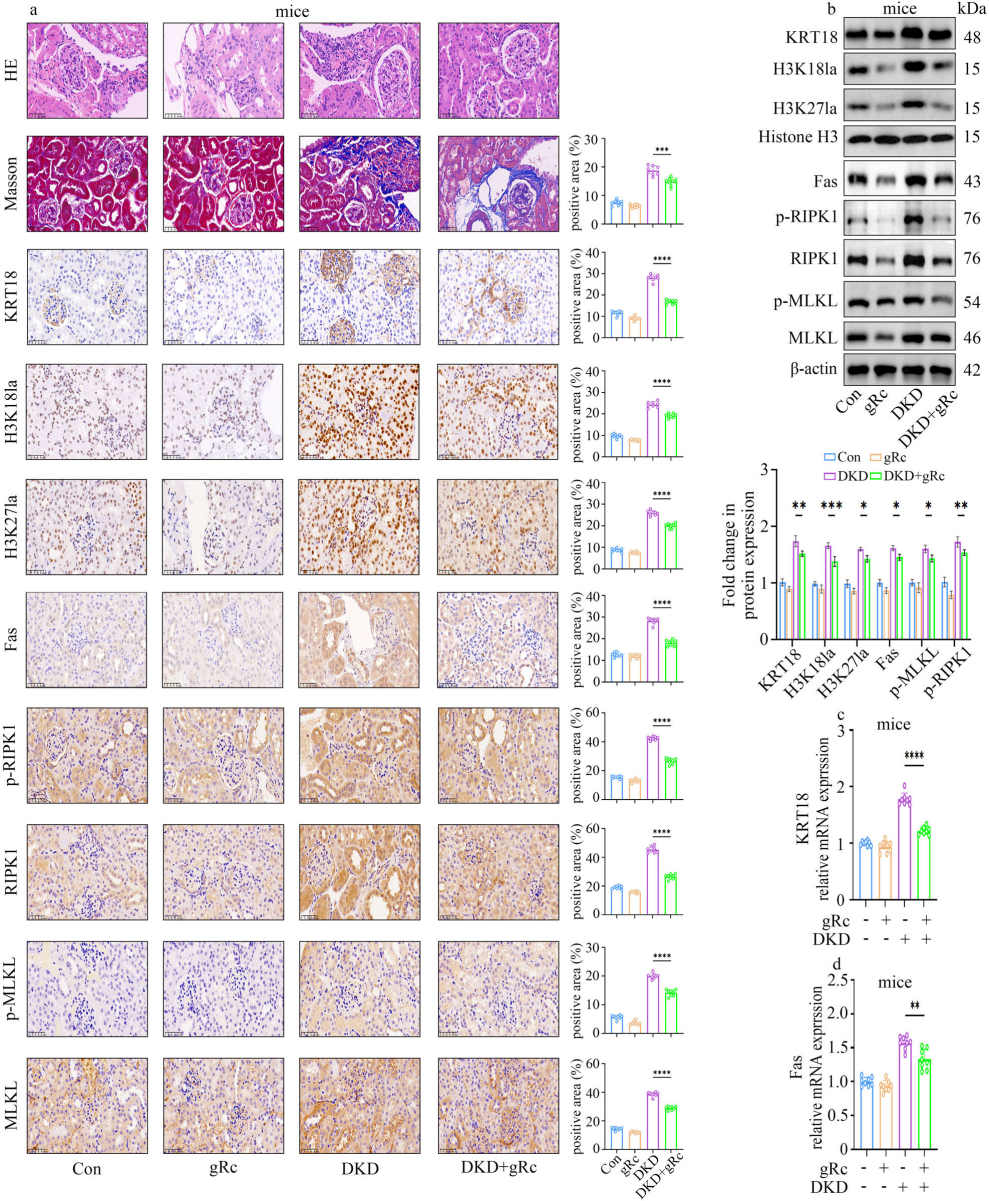
Ginsenoside Rc antagonizes KRT18-induced H3K18la and H3K27la expression to inhibit Fas transcription and necroptosis in diabetic kidney disease model mice. **a** Representative images of hematoxylin and eosin (HE), Masson’s trichrome staining, and immunohistochemistry staining for KRT18, H3K18la, H3K27la, Fas, p-RIPK1, RIPK1, p-MLKL, and MLKL in renal biopsy samples from control (Con), ginsenoside Rc (gRc), DKD, and DKD + gRc model mice (scale bar, 50 μm). The levels of KRT18, H3K18la, H3K27la, Fas, p-RIPK1, RIPK1, p-MLKL, and MLKL were increased in the kidneys of DKD model mice. Moreover, gRc treatment resulted in reduced levels of KRT18, H3K18la, H3K27la, and Fas; mitigated necroptosis; and attenuated renal injury (data are presented as the mean ± SD; *n* = 8 per group). **b** Western blotting revealed that the protein levels of KRT18, H3K18la, H3K27la, Fas, p-RIPK1, RIPK1, p-MLKL, and MLKL were increased in the kidneys of DKD model mice. Moreover, gRc treatment resulted in reduced levels of H3K18la, H3K27la, and Fas and mitigated necroptosis in DKD model mice. **c** Quantitative PCR assays revealed that KRT18 mRNA levels were increased in the kidneys of DKD model mice, but these changes were reversed by gRc treatment (data are presented as the mean ± SD; *n* = 8 per group). **d** Quantitative PCR assays revealed that the Fas mRNA levels were increased in the kidneys of DKD model mice, but these changes were reversed by gRc treatment (data are presented as the mean ± SD; *n* = 8 per group). *P < 0.05 and **P < 0.01.

Considering the pivotal role of KRT18-induced H3K18la and H3K27la in necroptosis in DKD, inhibiting KRT18-mediated histone lactylation may serve as a promising therapeutic strategy for DKD. Our aforementioned experimental findings (Fig. 5e, h and Supplementary Fig. 16a) confirmed that the ASN322, SER323, and GLU326 residues are essential for the lactylation activity of KRT18. We subsequently conducted molecular docking analyses of the structure of the pocket of KRT18 bound to small-molecule compounds from the HY-L022P FDA-Approved Drug Library Plus database, and the results indicated that gRc can bind to the same sites (Supplementary Fig. 19a). Moreover, ITC analysis demonstrated that the binding efficiency of gRc with KRT18 was greater than that of lactate (Supplementary Fig. 19b,c). Prior studies have indicated that gRc may function as a potential therapeutic agent for metabolic syndrome^36^, although the precise mechanisms underlying these effects remain to be fully elucidated. We propose that gRc suppresses the lactyltransferase activity of KRT18, thereby inhibiting Fas transcription and necroptosis and ultimately ameliorating DKD. Data from our in vitro lactylation experiments revealed that gRc treatment effectively inhibited KRT18-induced H3K18la and H3K27la expression in a concentration-dependent manner (Supplementary Fig. 19d). These results suggest that gRc may mitigate necroptosis in DKD by suppressing KRT18-mediated histone lactylation. Consequently, gRc was used to treat DKD models in this study. Our findings revealed that gRc treatment reduced the expression levels of KRT18, H3K18la, H3K27la, and Fas and suppressed necroptosis in hyperglycemic cells (Supplementary Fig. 20). More significantly, gRc treatment also decreased the levels of KRT18, H3K18la, H3K27la, and Fas (Fig. 7a–d), mitigated necroptosis and renal injury (Fig. 7a, b), and reduced serum BUN and Scr levels in DKD model mice (Supplementary Table 5).

### gRc can effectively alleviate myocardial injury in diabetic mice

**Fig. 8:**
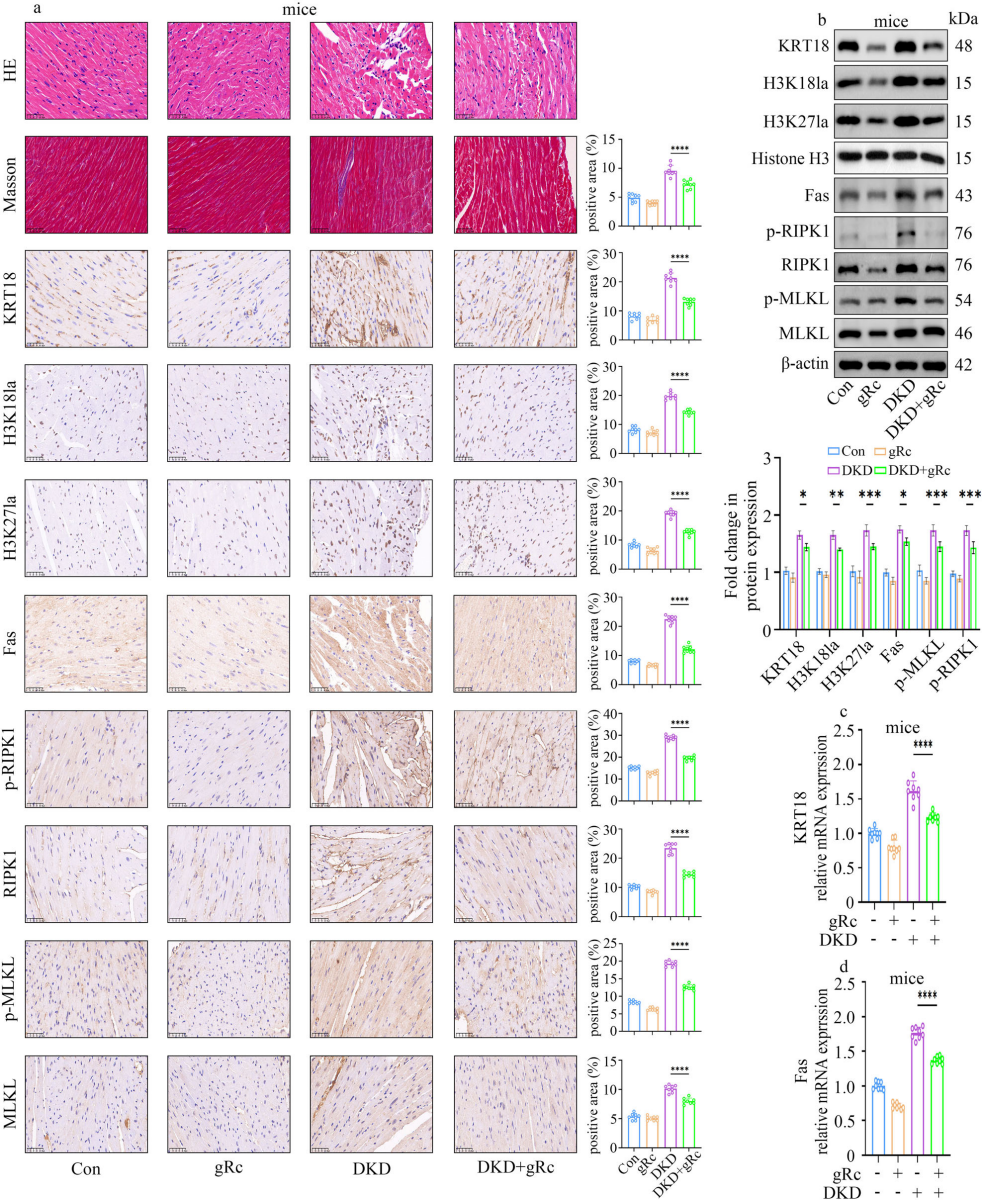
Ginsenoside Rc effectively alleviates cardiac injury in diabetic kidney disease model mice. **a** Representative microscopy images showing hematoxylin and eosin (HE) staining, Masson’s trichrome staining, and immunohistochemistry staining of cardiac tissue specimens from the control (Con), ginsenoside Rc (gRc), diabetic kidney disease (DKD), and DKD + gRc groups of mice (scale bar, 50 μm). gRc treatment reduced cardiac damage and interstitial fibrosis; decreased the expression levels of KRT18, H3K18la, H3K27la, and Fas; and inhibited necroptosis in the hearts of DKD model mice (data are presented as the mean ± SD; *n* = 8 per group). **b** Western blotting analysis revealed that gRc treatment decreased KRT18 expression; reduced the protein levels of H3K18la, H3K27la, and Fas; and inhibited necroptosis in the hearts of DKD model mice. **c** Quantitative PCR indicated that gRc treatment decreased KRT18 mRNA expression in the hearts of DKD model mice (data are presented as the mean ± SD; *n* = 8 per group). **d** Quantitative PCR indicated that gRc treatment decreased Fas mRNA expression in the hearts of DKD model mice (data are presented as the mean ± SD; *n* = 8 per group). *P < 0.05 and **P < 0.01.

A significant epidemiological association between diabetes and myocardial injury has been identified^37^. Additionally, histone lactylation has a crucial role in diabetic myocardial injury^38^. On the basis of these findings, we further explored whether gRc improves diabetic myocardial injury by inhibiting KRT18-mediated lactylation. Our data indicated that gRc treatment decreased KRT18, H3K18la, H3K27la, and Fas levels and alleviated necroptosis and cardiac injury in DKD model mice (Fig. 8). These data suggest that inhibiting KRT18-induced histone lactylation by gRc treatment might be an efficacious therapeutic strategy for diabetes-related complications, including DKD and diabetic myocardial injury.

## Discussion

The main finding of the present study was that hyperglycemia-mediated H3K18la and H3K27la expression triggers necroptosis by modulating Fas transcription, thus contributing to DKD. Moreover, we determined that KRT18 acts as a lactyltransferase to modulate H3K18la and H3K27la, thus triggering Fas transcription and necroptosis in DKD. In addition, the inhibition of KRT18-induced H3K18la and H3K27la expression by gRc treatment effectively alleviated Fas transcription and necroptosis, thus attenuating renal dysfunction in DKD models.

DKD represents a prevalent microvascular complication of diabetes and has emerged as the leading cause of end-stage renal disease globally^1,2^. The pathogenesis of DKD involves multiple intricate pathophysiological processes, including oxidative stress, cell death, epigenetic modulation, and hemodynamic alterations^39–41^. Among these mechanisms, cell death, which acts as a pivotal component, has garnered increasing attention in recent years and is recognized as one of the direct pathological drivers contributing to DKD^7^. Hyperglycemia can induce necroptosis in cells and significantly contributes to the pathogenesis and progression of diabetes and its complications^42–44^. Elevated levels of hyperglycemia^45^ and advanced glycation end products^46^ have been shown to induce necroptosis in cardiomyocytes, thereby facilitating the development of diabetic myocardial fibrosis. Furthermore, in patients with diabetes, hyperglycemia-induced necroptosis of endothelial cells may accelerate the formation of atherosclerotic plaques^47^, whereas necroptosis of podocytes^44^ and glomerular endothelial cells^43^ is strongly associated with diabetic glomerular lesions and fibrosis. In this study, genetic ablation of MLKL in a DKD mouse model led to significant improvements in renal dysfunction and a marked attenuation of renal fibrosis. Collectively, these findings indicate that necroptosis has a critical role in the pathophysiological process of DKD. Next, we investigated the potential mechanisms by which necroptosis occurs in DKD. By analyzing the RNA-seq and CUT&Tag data, we determined that Fas might be involved in necroptosis in DKD. Fas/FasL signaling has a crucial role in triggering necroptosis^10,11^. However, whether Fas participates in DKD is still not well known. By silencing Fas, we confirmed that Fas has a significant role in necroptosis in hyperglycemic cells. Hence, further exploration of the transcriptional mechanism of Fas in DKD may provide novel insights for new therapeutic strategies.

Epigenetic modifications are now recognized as critical regulators of the development of diabetes-associated complications^15^. Accumulating data indicate that lactate-induced histone lactylation, a newly discovered epigenetic mechanism, contributes to the pathophysiological processes of DKD^19,20,48,49^. In this study, we observed that H3K18la and H3K27la levels gradually increased as the DKD stage progressed. In addition, the suppression of H3K18la and H3K27la expressions using OXA or STP mitigated necroptosis, leading to reduced cell death and the amelioration of renal dysfunction in DKD models. Moreover, lactate treatment increased necroptosis, thus increasing cell death and aggravating renal dysfunction in DKD models. Furthermore, the effects of HG levels on increasing Fas expression and necroptosis were significantly attenuated or abolished after the overexpression of the H3-K18/27R mutant in cells. Our findings suggest that hyperglycemia-driven H3K18la and H3K27la are involved in the pathogenesis of DKD and that targeting lactate accumulation or modulating H3K18la and H3K27la levels could represent promising therapeutic strategies for DKD.

Although DKD has substantial clinical significance, the roles of lactylation regulatory proteins in this condition remain largely uninvestigated. There is an urgent need to identify potential lactyltransferases and delactyltransferases to facilitate the development of novel therapeutic strategies for DKD. Recently, a group of enzymes involved in lactylation, such as p300, AARS1, sirtuins 1–3, and histone deacetylases 1–3, have been reported^21–23^. Furthermore, some of these enzymes have been confirmed to be involved in the pathological process of DKD and are regarded as potential therapeutic targets. It has been reported that p300-mediated lactylation of TRIM65 at lysine 206 compromises its dual regulatory functions in suppressing ferroptosis and glycolysis, thereby contributing to the progression of DKD^25^. Moreover, AARS1 lactylates H3K18 to modulate fatty acid elongase-5 and acyl-CoA synthetase long-chain family member 4 transcription, thus participating in DKD^26,27^. In addition, interventions targeting p300-mediated and AARS1-mediated lactylation modifications can ameliorate DKD^25–27^. However, our mass spectrometry data did not reveal any evidence suggesting interactions between these lactylation-modifying enzymes and H3K18la or H3K27la. These results indicate that known lactylation-modifying enzymes may not be the primary regulators of H3K18la and H3K27la in DKD. Through a further detailed analysis of the mass spectrometry findings, we identified members of the KRT family as interacting partners of H3K18la and H3K27la. Subsequent crystallographic studies and co-IP and GST pulldown assays confirmed that KRT18 directly interacts with H3. Moreover, molecular docking and ITC experiments revealed that KRT18 directly binds to lactate. In vitro lactylation assays further verified that KRT18 functions as a lactyltransferase responsible for the lactylation of H3K18 and H3K27. KRT18 participates in various cellular activities, such as providing resistance to external stress and preserving the structural integrity of the cytoplasm and mitochondria^29^. More recent studies have suggested that KRT18 performs its biological functions by regulating the transcription of genes^31^. In this study, we observed increased KRT18 expression in hyperglycemic cells and in the kidneys of DKD model mice and patients. Furthermore, the inhibition of KRT18 expression reduced H3K18la, H3K27la, and Fas levels; suppressed necroptosis; and alleviated renal injury in both DKD model mice and hyperglycemic cells. Conversely, KRT18 overexpression increased H3K18la, H3K27la, and Fas levels, thereby inducing necroptosis in cells. Notably, the mutant KRT18, which cannot interact with lactate, failed to produce these effects. Additionally, ChIP assays confirmed that H3K18la, H3K27la, and KRT18 were enriched at the promoter region of Fas. Moreover, KRT18 overexpression-induced necroptosis was reversed by silencing Fas in cells. Taken together, these findings suggest that KRT18 serves as a lactyltransferase to modify H3K18 and H3K27 lactylation, thereby regulating Fas transcription and participating in necroptosis in DKD.

Previous studies have indicated that gRc might be a potential therapeutic candidate for metabolic syndrome^36^. However, the exact mechanism underlying its effects remains largely unknown. Through molecular docking experiments, we revealed that gRc binds to KRT18 at a site highly similar to the binding site of lactate. Moreover, the ITC results revealed that the binding efficiency of gRc with KRT18 was greater than that of lactate. Additionally, in vitro lactylation assays demonstrated that gRc treatment significantly suppressed KRT18-mediated lactylation in a concentration-dependent manner. On the basis of these observations, we administered gRc to hyperglycemic cells and DKD model mice. Our findings revealed that gRc treatment effectively reduced the levels of KRT18, H3K18la, and H3K27la, thereby decreasing Fas transcription, inhibiting necroptosis, alleviating kidney injury, and ameliorating renal dysfunction in DKD models. Taken together, the results of our study suggest that gRc may serve as an effective therapeutic strategy for DKD by inhibiting KRT18-induced lactylation.

This study has several limitations that should be acknowledged. First, although LDHA was the focus of this investigation, other glycolytic enzymes may also contribute to lactate production and have a role in DKD; however, this possibility requires further exploration. Second, the results of the present study revealed that KRT18 functions as a lactyltransferase to modulate Fas transcription in DKD via the lactylation of H3K18 and H3K27; whether other lactyltransferases or delactyltransferases participate in the modulation of FAS transcription in DKD warrants further exploration. Third, the role of lactate-mediated non-histone lactylation in DKD was not fully elucidated in this study and deserves further investigation. Fourth, to further verify whether increasing H3K18la and H3K27la would exacerbate necroptosis in DKD, lactate was used to treat DKD model mice. Whether lactate administration simultaneously affects other pathways associated with lactate metabolism deserves further research. Fifth, the results of the IHC assay indicated that the expression of p-MLKL and p-RIPK1 was increased in both the glomeruli and renal tubules of patients and model mice with DKD. These findings suggest that necroptosis occurs in both glomerular endothelial cells and renal tubular epithelial cells. However, which type of renal cell necroptosis contributes primarily to the pathogenesis of DKD was not clarified in this study and deserves further research.

In conclusion, this study revealed that KRT18 serves as a lactyltransferase to modulate H3K18la and H3K27la, which subsequently activates Fas transcription and induces necroptosis in DKD. Moreover, the suppression of KRT18-mediated lactylation by gRc could represent a potential and efficient therapeutic approach for the treatment of DKD.

## Supplementary information

Supplementary Information

Supplementary Tables

## Data Availability

The data sets used and/or analyzed during the current study are available from the corresponding author upon reasonable request.
