# Supplementary material for: Keratin 18 functions as a lactyltransferase to trigger necroptosis in diabetic kidney disease by modulating Fas transcription
Source: Exp Mol Med. 2026 Jun 12;58(6):2007–23. doi: 10.1038/s12276-026-01737-9 (PMC13323999; doi:10.1038/s12276-026-01737-9)
Supplement: Supplementary file 1 — Supplementary Information [file 12276_2026_1737_MOESM1_ESM.pdf]

Supplementary Materials

Supplementary Figure 1-20

Supplementary Table 1-15

Supplementary Figures

Supplementary Fig. 1. The levels of lactate, lysine lactylation (Kla), H3K18la and H3K27la, and cell death are increased in hyperglycemic cells

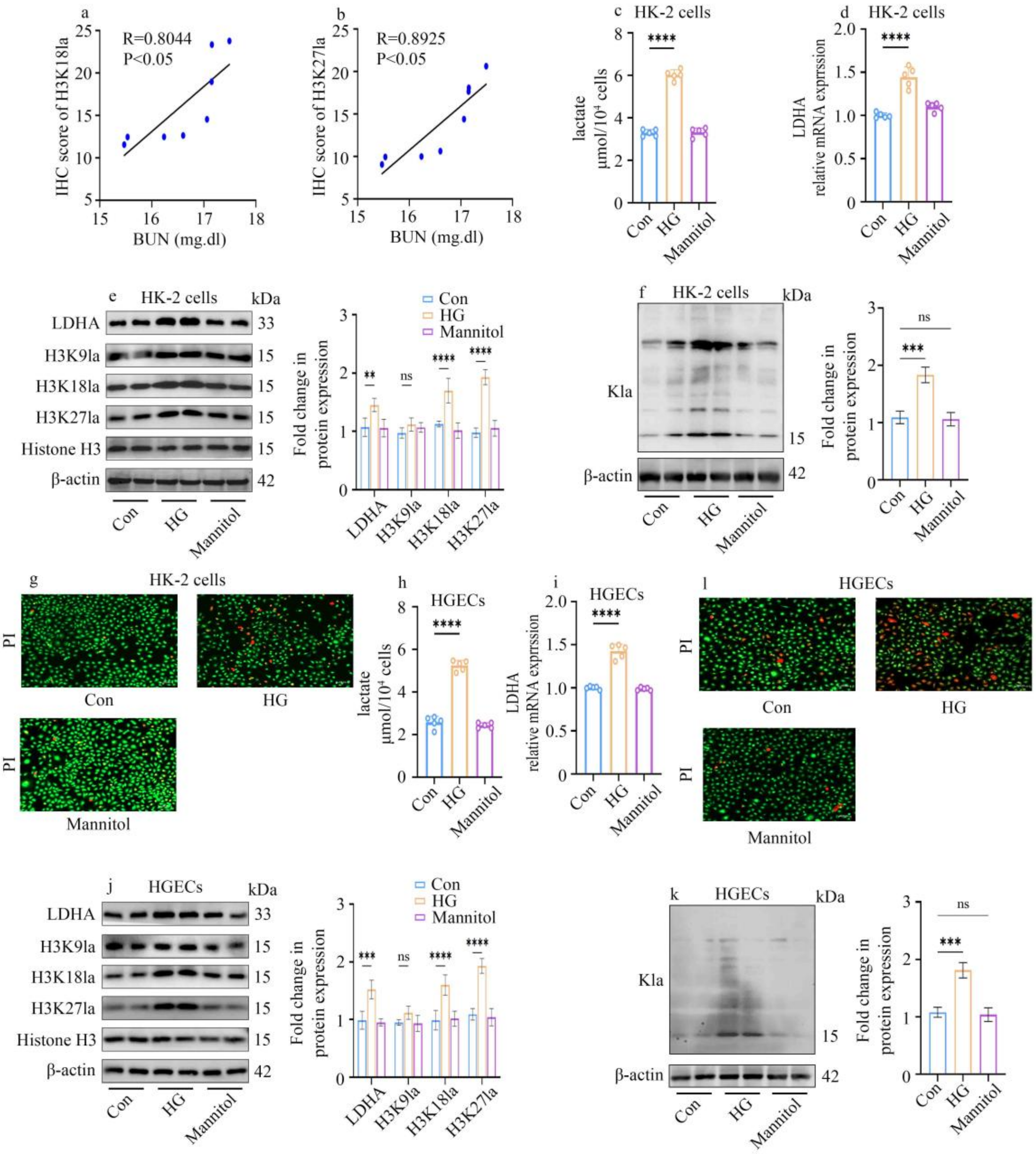

(a) The expression of H3K18la was positively correlated with BUN levels in DKD model mice. (b) The expression of H3K27la was positively correlated with BUN levels in DKD model mice. (c) Lactate levels were increased in HK-2 cells treated with high concentrations of glucose (data are presented as the mean ±SD; n=5/group).

(d) qPCR assays revealed that LDHA mRNA expression was upregulated in HK-2 cells treated with high concentrations of glucose (data are presented as the mean ±SD; n=5/group). (e) Western blotting revealed that the protein levels of LDHA, H3K18la and H3K27la increased in HK-2 cells treated with high concentrations of glucose; however, the level of H3K9la did not significantly change. (f) Western blotting revealed that the protein level of Kla increased in HK-2 cells treated with high concentrations of glucose. (g) The death of HK-2 cells treated with high concentrations of glucose increased. (h) Lactate levels were increased in HGECs treated with high concentrations of glucose (data are presented as the mean ±SD; n=5/group). (i) qPCR assays revealed that LDHA mRNA expression was upregulated in HGECs treated with high concentrations of glucose (data are presented as the mean ±SD; n=5/group). (j) Western blotting revealed that the protein levels of LDHA, H3K18la and H3K27la increased in HGECs treated with high concentrations of glucose; however, the level of H3K9la did not significantly change. (k) Western blotting revealed that the protein level of Kla increased in HGECs treated with high concentrations of glucose. (l) The death of HGECs treated with high concentrations of glucose increased. *P<0.05 and **P<0.01.

Supplementary Fig. 2. The suppression of lactate accumulation and reduction in H3K18la and H3K27la levels alleviates cell death and renal dysfunction in diabetic kidney disease (DKD) model mice

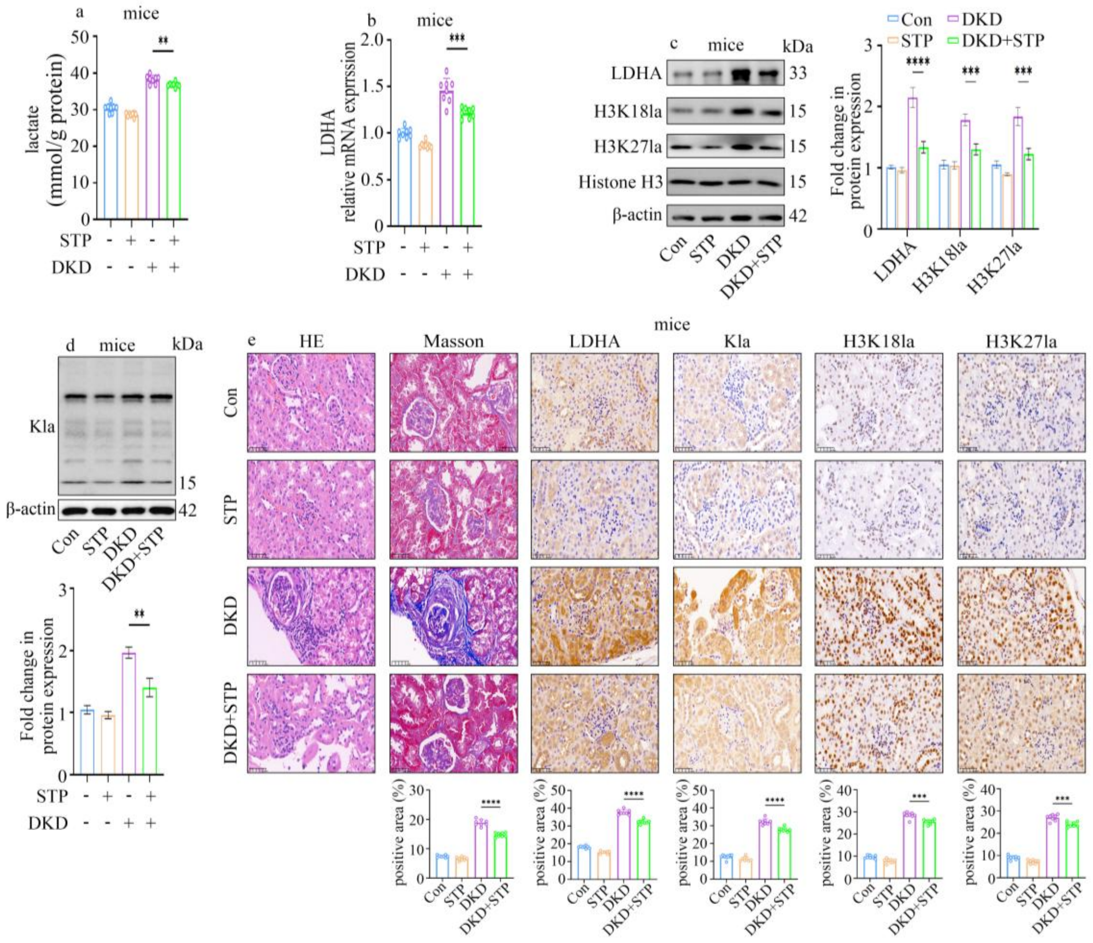

(a) Stiripentol (STP) treatment decreased lactate accumulation in the renal tissues of DKD model mice (data are presented as the mean ±SD; n=8/group). (b) STP treatment decreased LDHA mRNA levels in the renal tissues of DKD model mice (data are presented as the mean ±SD; n=8/group). (c) STP treatment decreased LDHA, H3K18la and H3K27la levels in the renal tissues of DKD model mice. (d) STP treatment reduced lysine lactylation (Kla) levels in the renal tissues of DKD model mice. (e) STP treatment attenuated renal injury and fibrosis and decreased LDHA, Kla, H3K18la and H3K27la levels in the renal tissues of DKD model mice (data are

presented as the mean ±SD; n=8/group). *P<0.05 and **P<0.01.

Supplementary Fig. 3. Suppressing lactate accumulation and reducing H3K18la and H3K27la levels via oxamate (OXA) reduces the death of hyperglycemic HGECs

(a) OXA treatment decreased lactate accumulation in HGECs treated with high concentrations of glucose (data are presented as the mean ±SD; n=5/group). (b) OXA treatment decreased LDHA mRNA levels in HGECs treated with high concentrations of glucose (data are presented as the mean ±SD; n=5/group). (c) OXA treatment decreased LDHA, H3K18la and H3K27la levels in HGECs treated with high concentrations of glucose. (d) OXA treatment reduced Kla levels in HGECs treated with high concentrations of glucose. (e) OXA treatment decreased the death of HGECs treated with high concentrations of glucose. *P<0.05 and **P<0.01.

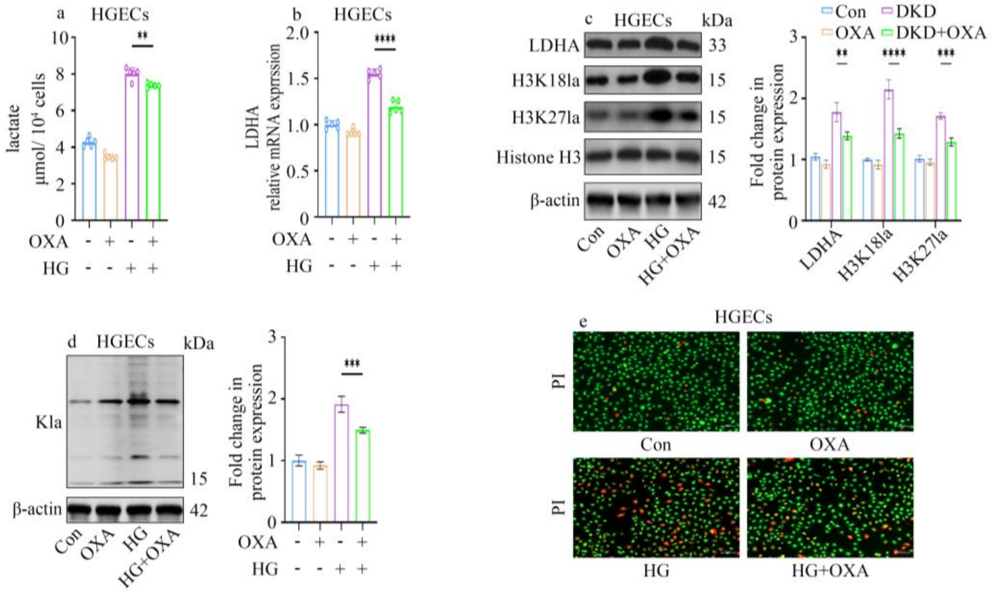

Supplementary Fig. 4. The suppression of lactate accumulation and reduction in H3K18la and H3K27la levels induced by stiripentol (STP) reduces the death of hyperglycemic cells

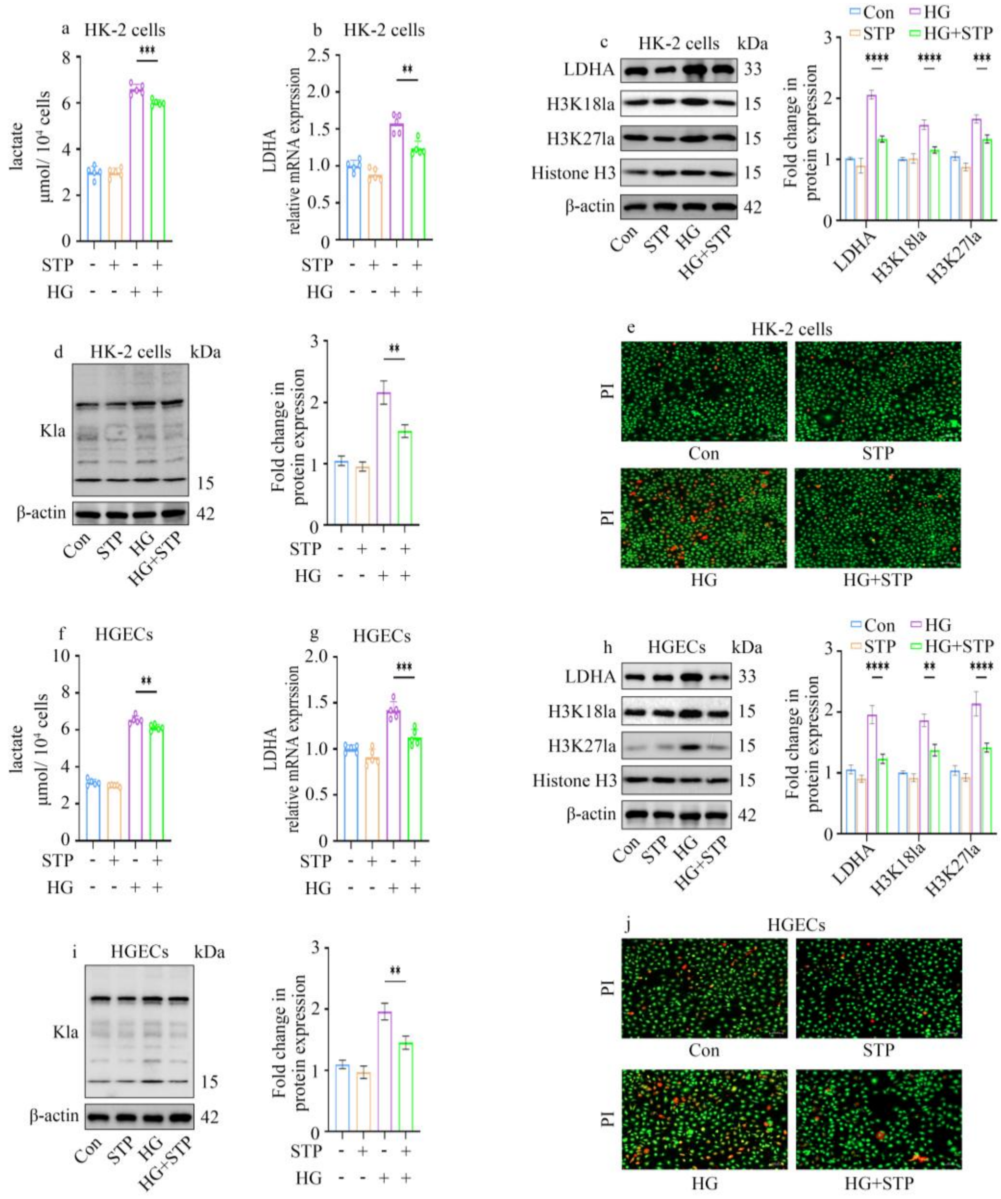

(a) STP treatment decreased lactate accumulation in HK-2 cells treated with high concentrations of glucose (data are presented as the mean ±SD; n=5/group). (b) STP treatment decreased LDHA mRNA levels in HK-2 cells treated with high concentrations of glucose (data are presented as the mean ±SD; n=5/group). (c) STP treatment decreased LDHA, H3K18la and H3K27la levels in HK-2 cells treated with

high concentrations of glucose. (d) STP treatment reduced lysine lactylation (Kla) levels in HK-2 cells treated with high concentrations of glucose. (e) STP treatment decreased the death of HK-2 cells treated with high concentrations of glucose. (f) STP treatment decreased lactate accumulation in HGECs treated with high concentrations of glucose (data are presented as the mean ±SD; n=5/group). (g) STP treatment decreased LDHA mRNA levels in HGECs treated with high concentrations of glucose (data are presented as the mean ±SD; n=5/group). (h) STP treatment decreased LDHA, H3K18la and H3K27la levels in HGECs treated with high concentrations of glucose. (i) STP treatment reduced Kla levels in HGECs treated with high glucose concentrations. (j) STP treatment decreased the death of HGECs treated with high glucose concentrations. *$P<0.05$ and **$P<0.01$.

Supplementary Fig. 5. Increased lactate accumulation and increased H3K18la and H3K27la levels caused by lactate treatment exacerbate cell death and renal dysfunction in diabetic kidney disease (DKD) model mice

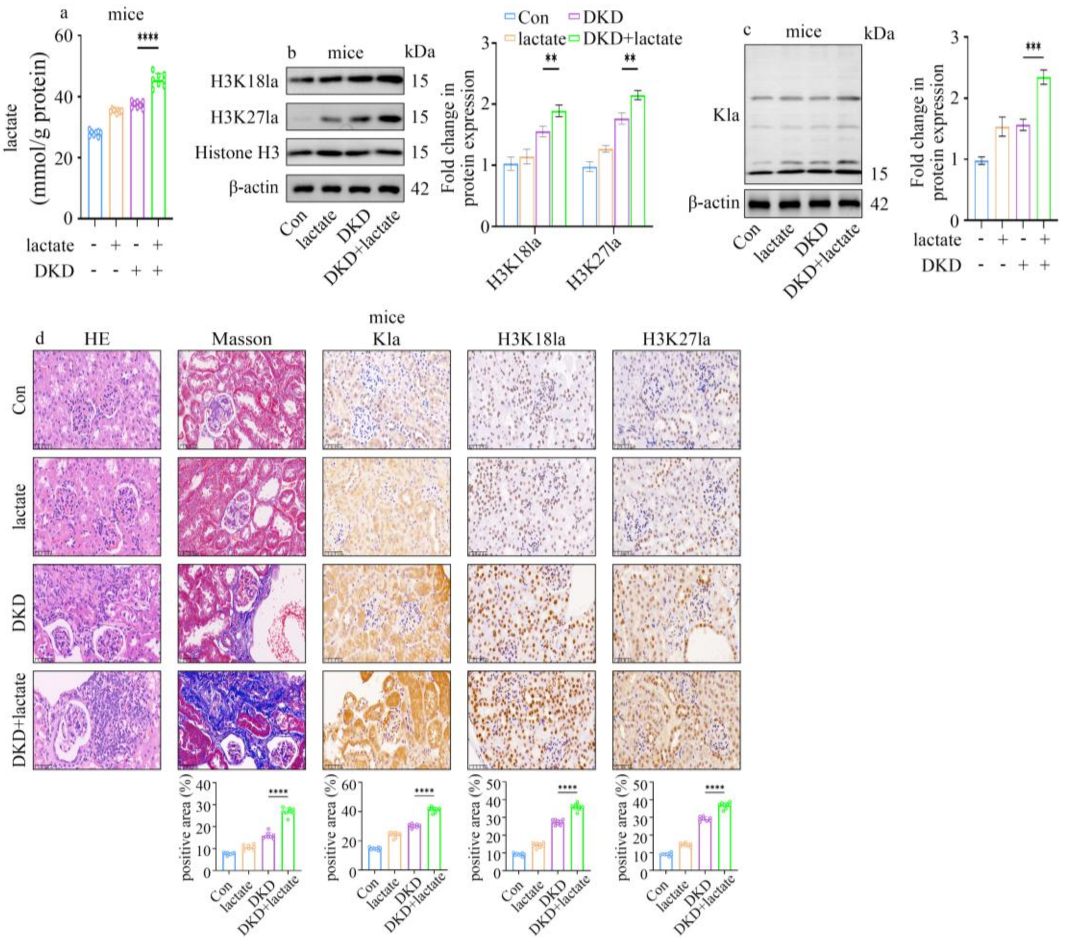

(a) Lactate treatment increased lactate accumulation in the renal tissues of DKD model mice (data are presented as the mean ±SD; n=8/group). (b) Lactate treatment increased H3K18la and H3K27la levels in the renal tissues of DKD model mice. (c) Lactate treatment increased lysine lactylation (Kla) levels in the renal tissues of DKD model mice. (d) Lactate treatment aggravated renal injury and fibrosis and increased Kla, H3K18la and H3K27la levels in the renal tissues of DKD model mice (data are presented as the mean ±SD; n=8/group). *P<0.05 and **P<0.01.

Supplementary Fig. 6. Increased lactate accumulation and H3K18la and H3K27la levels due to lactate treatment exacerbate the death of hyperglycemic cells

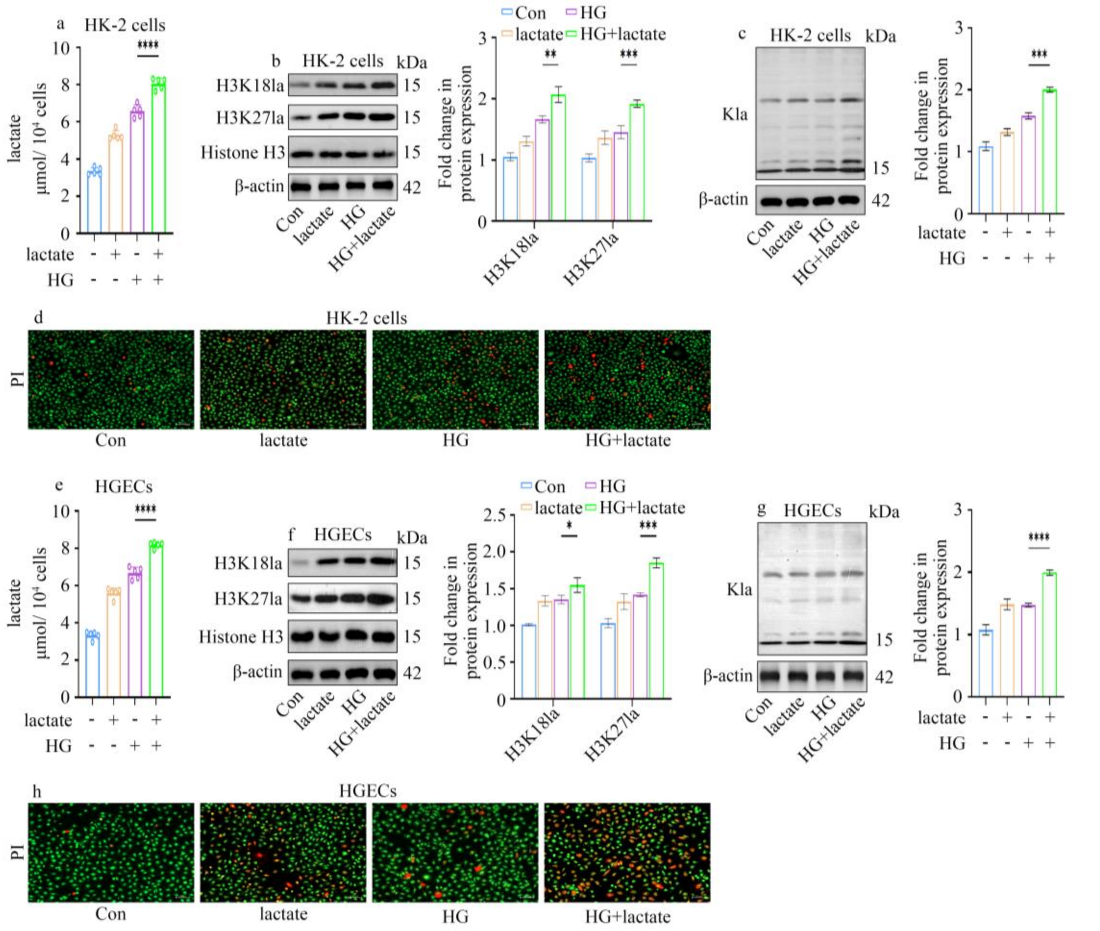

(a) Lactate treatment increased lactate accumulation in HK-2 cells treated with high concentrations of glucose (data are presented as the mean ±SD; n=5/group). (b) Lactate treatment increased H3K18la and H3K27la levels in HK-2 cells treated with high concentrations of glucose. (c) Lactate treatment increased lysine lactylation (Kla) levels in HK-2 cells treated with high concentrations of glucose. (d) Lactate treatment increased the death of HK-2 cells treated with high concentrations of glucose. (e) Lactate treatment increased lactate accumulation in HGECs treated with high concentrations of glucose (data are presented as the mean ±SD; n=5/group). (f) Lactate treatment increased H3K18la and H3K27la levels in HGECs treated with high

concentrations of glucose. (g) Lactate treatment increased Kla levels in HGECs treated with high concentrations of glucose. (h) Lactate treatment increased the death of HGECs treated with high concentrations of glucose. *$P<0.05$ and **$P<0.01$.

Supplementary Fig. 7. Lactate-induced H3K18la and H3K27la expression triggers the death of hyperglycemic cells, as well as LDHA transcription

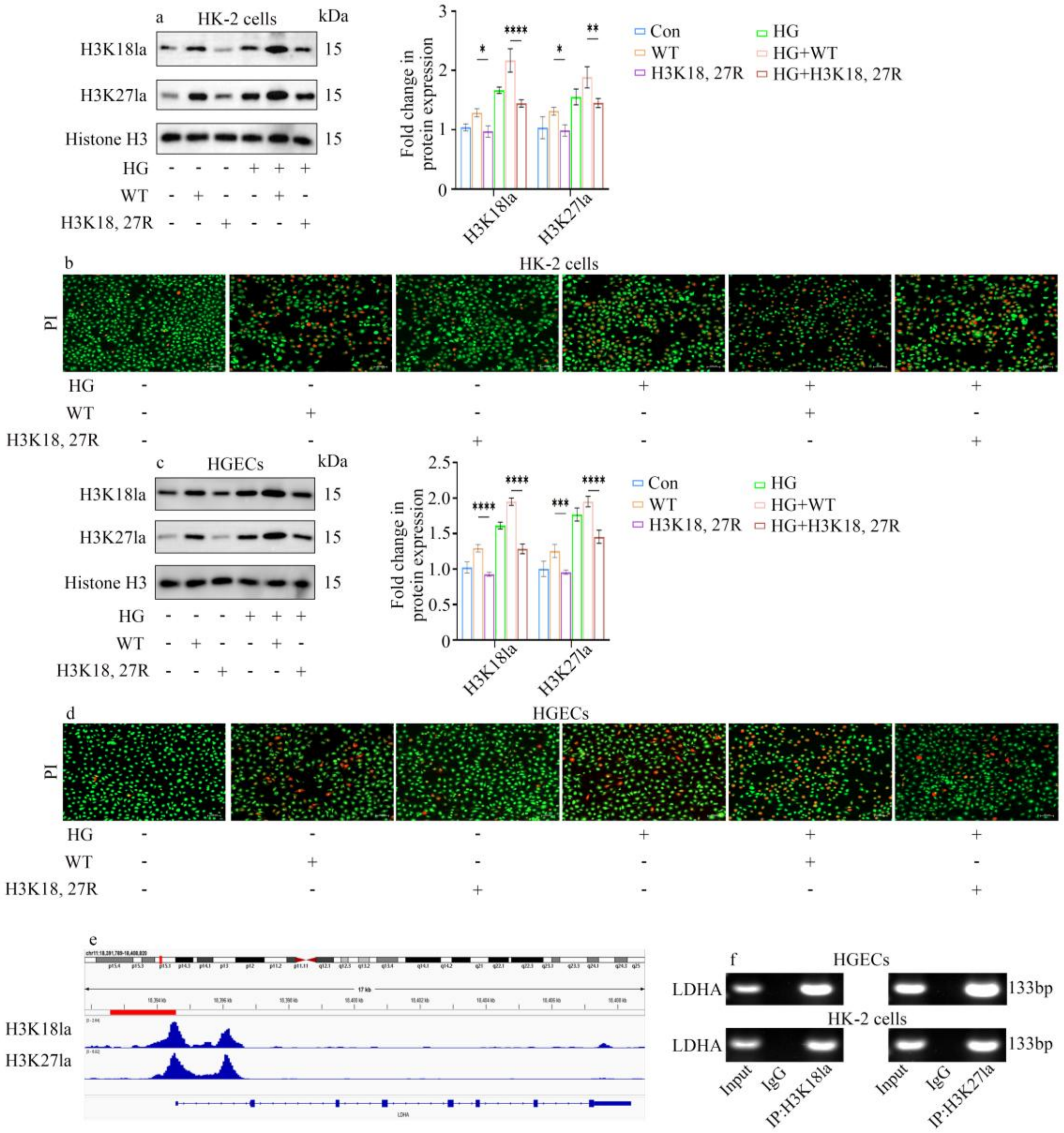

(a) The expression of the H3-K18/27R mutant significantly reduced H3K18la and H3K27la levels following high-glucose treatment compared with those in the H3-WT group of HK-2 cells. (b) The expression of the H3-K18/27R mutant in HK-2 cells significantly attenuated cell death following high-glucose treatment compared with that in the H3-WT group of HK-2 cells. (c) The expression of the H3-K18/27R mutant significantly reduced H3K18la and H3K27la levels following high-glucose treatment

compared with the levels in the H3-WT group of HGECs. (d) The expression of the H3-K18/27R mutant significantly attenuated cell death following high-glucose treatment compared with that in the H3-WT group of HGECs. (e) CUT&Tag data indicating that H3K18la and H3K27la may occupy the promoter region of LDHA. (f) ChIP assays verified that H3K18la and H3K27la occupied the promoter region of LDHA in cells. *$P<0.05$ and **$P<0.01$.

Supplementary Fig. 8. Lactate-induced H3K18la and H3K27la expression triggers necroptosis in hyperglycemic cells

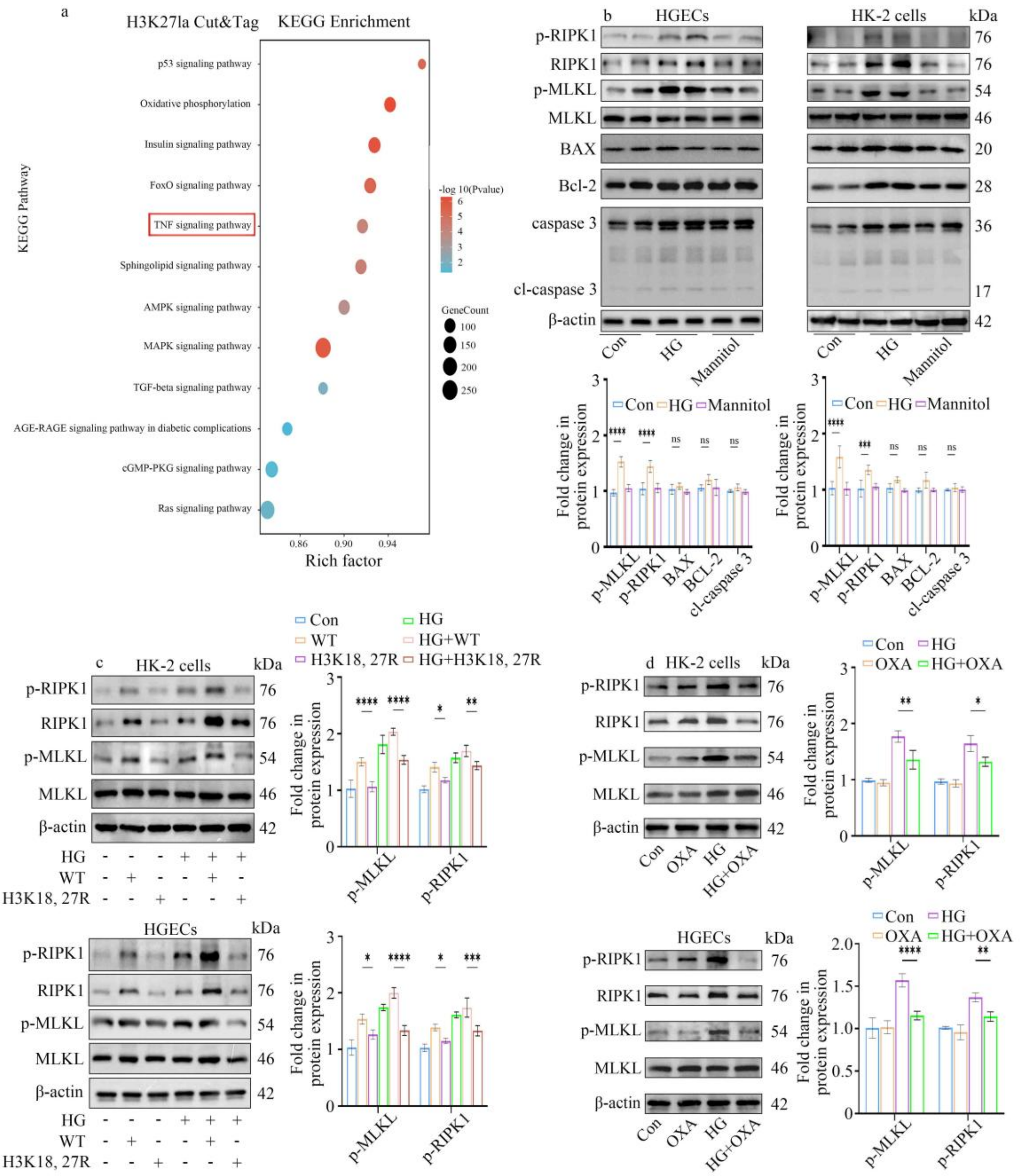

(a) Analysis of the molecular function of H3K27la using CUT&Tag data and the Kyoto Encyclopedia of Genes and Genomes (KEGG) database. (b) Western blotting indicated that necroptosis induced the death of hyperglycemic cells. (c) Compared with the H3-WT, the H3-K18/27R mutation significantly inhibited necroptosis in cells following high-glucose treatment. (d) Oxamate (OXA) treatment inhibited necroptosis

in hyperglycemic cells.

Supplementary Fig. 9. Stiripentol (STP) treatment inhibits necroptosis in DKD models

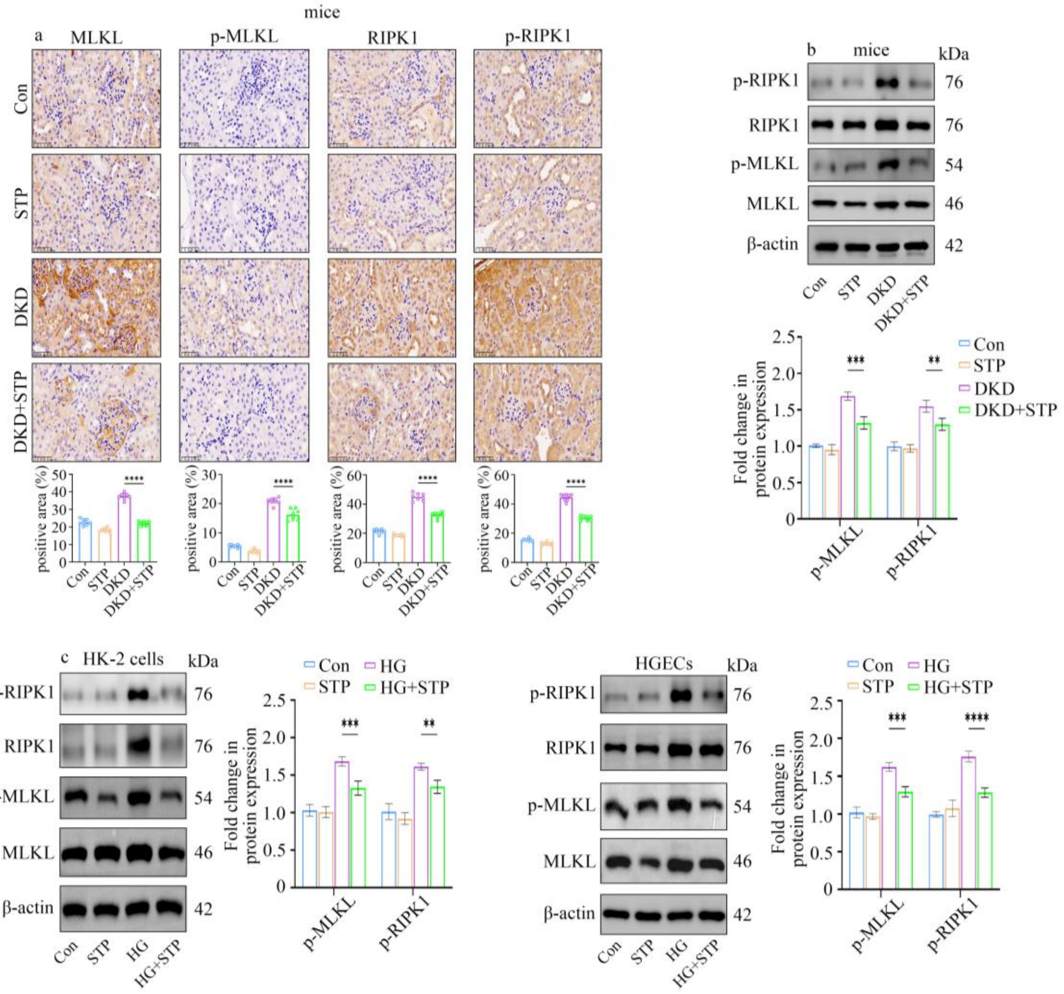

(a) Immunohistochemical (IHC) staining indicated that STP treatment decreased p-RIPK1 and p-MLKL levels in the kidneys of DKD model mice (data are presented as the mean ±SD; n=8/group). (b) Western blotting indicated that STP treatment decreased p-RIPK1 and p-MLKL levels in the kidneys of DKD model mice. (c) Western blotting indicated that STP treatment decreased p-RIPK1 and p-MLKL levels in hyperglycemic cells. *P<0.05 and **P<0.01.

Supplementary Fig. 10. Lactate treatment aggravates necroptosis in DKD models

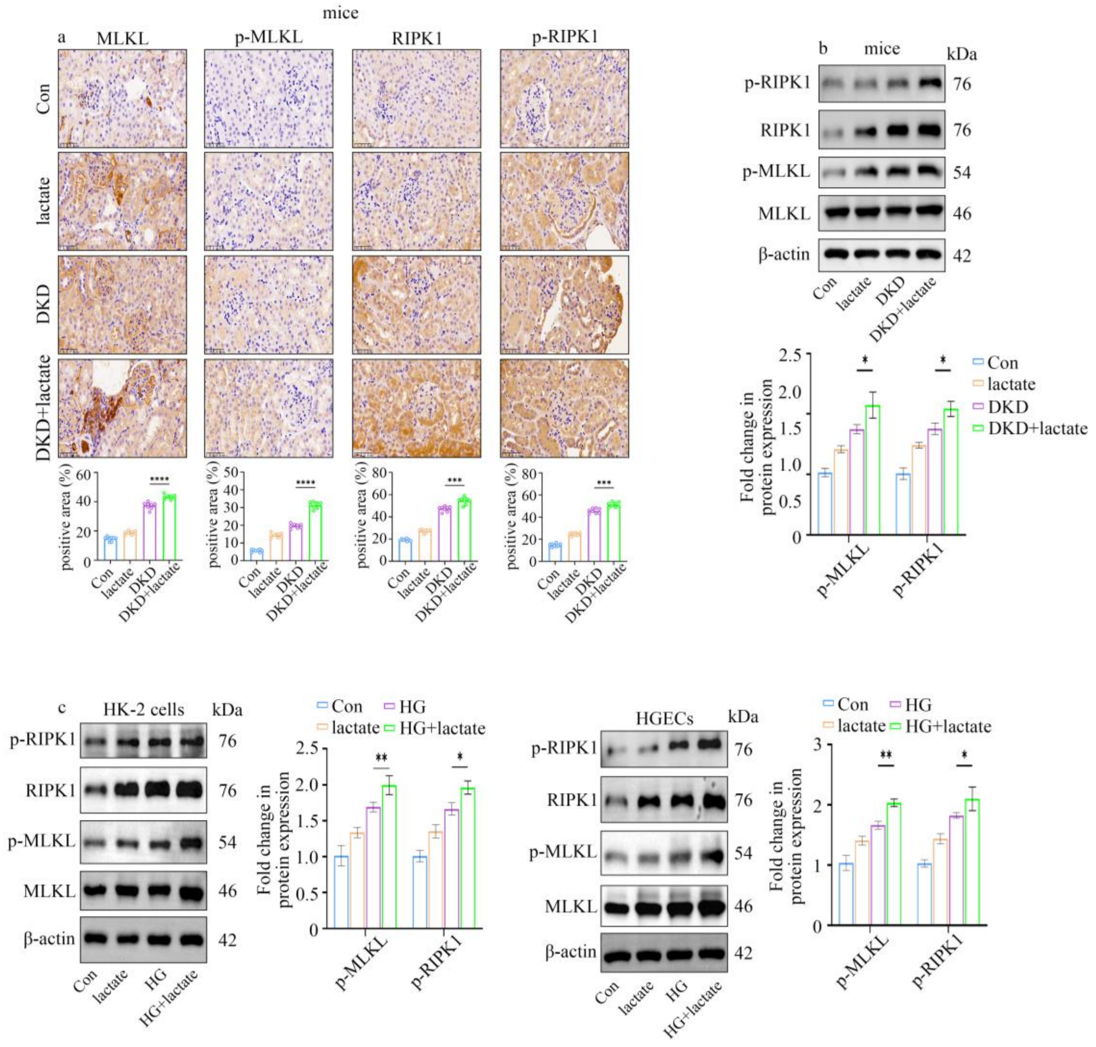

(a) Immunohistochemical (IHC) staining indicated that lactate treatment increased p-RIPK1 and p-MLKL levels in the kidneys of DKD model mice (data are presented as the mean ±SD; n=8/group). (b) Western blotting indicated that lactate treatment increased p-RIPK1 and p-MLKL levels in the kidneys of DKD model mice. (c) Western blotting indicated that lactate treatment increased p-RIPK1 and p-MLKL levels in hyperglycemic cells. *P<0.05 and **P<0.01.

Supplementary Fig. 11. Fas expression increases in hyperglycemic cells but decreases with oxamate (OXA) treatment

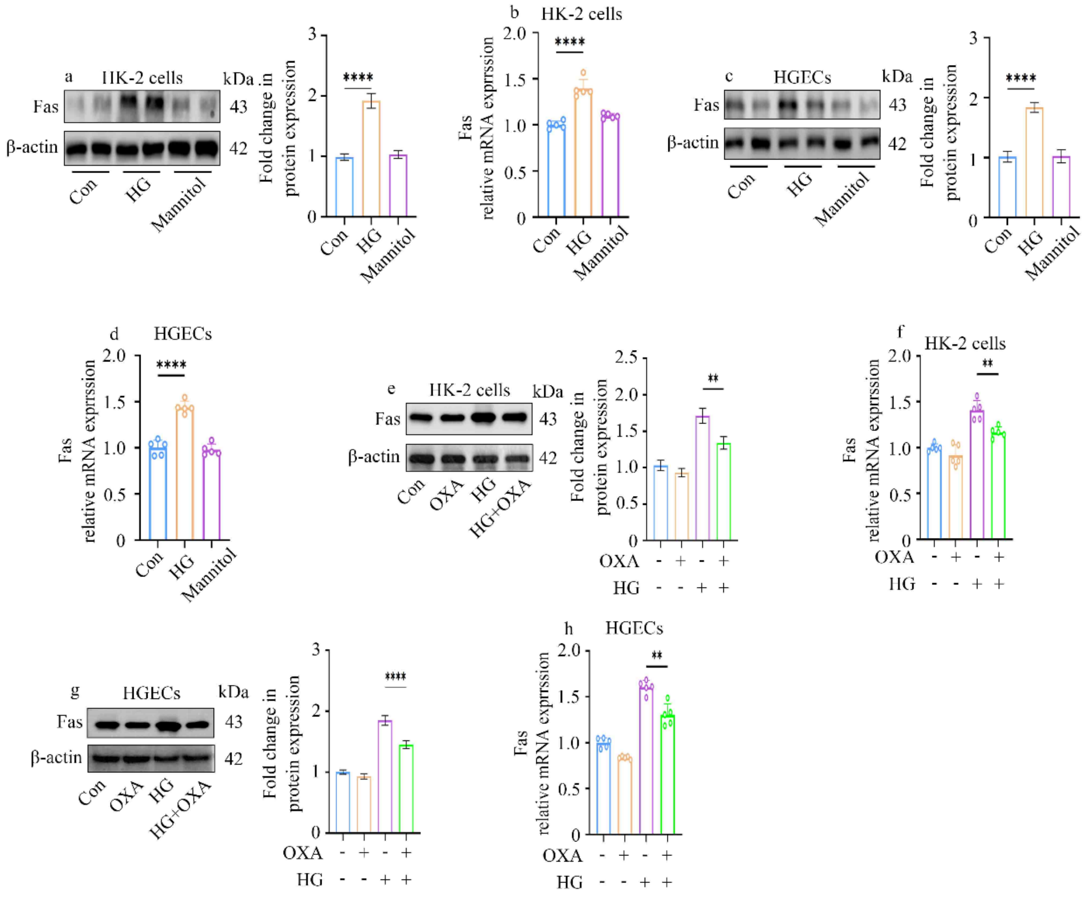

(a) Western blotting indicated that Fas protein expression was increased in HK-2 cells treated with high concentrations of glucose. (b) The results of the qPCR assay indicated that Fas mRNA expression was increased in HK-2 cells treated with high concentrations of glucose (data are presented as the mean ±SD; n=5/group). (c) Western blotting indicated that Fas protein expression was increased in HGECs treated with high concentrations of glucose. (d) qPCR indicated that Fas mRNA expression was increased in HGECs treated with high concentrations of glucose (data are presented as the mean ±SD; n=5/group). (e) Western blotting indicated that OXA treatment decreased Fas protein expression in HK-2 cells treated with high

concentrations of glucose. (f) qPCR indicated that OXA treatment decreased Fas mRNA expression in HK-2 cells treated with high concentrations of glucose (data are presented as the mean ±SD; n=5/group). (g) Western blotting indicated that OXA treatment decreased Fas protein expression in HGECs treated with high concentrations of glucose. (h) qPCR indicated that OXA treatment decreased Fas mRNA expression in HGECs treated with high concentrations of glucose (data are presented as the mean ±SD; n=5/group). $*P<0.05$ and $**P<0.01$.

Supplementary Fig. 12. Fas expression in the kidneys of DKD model mice is modulated by treatment with stiripentol (STP) or lactate

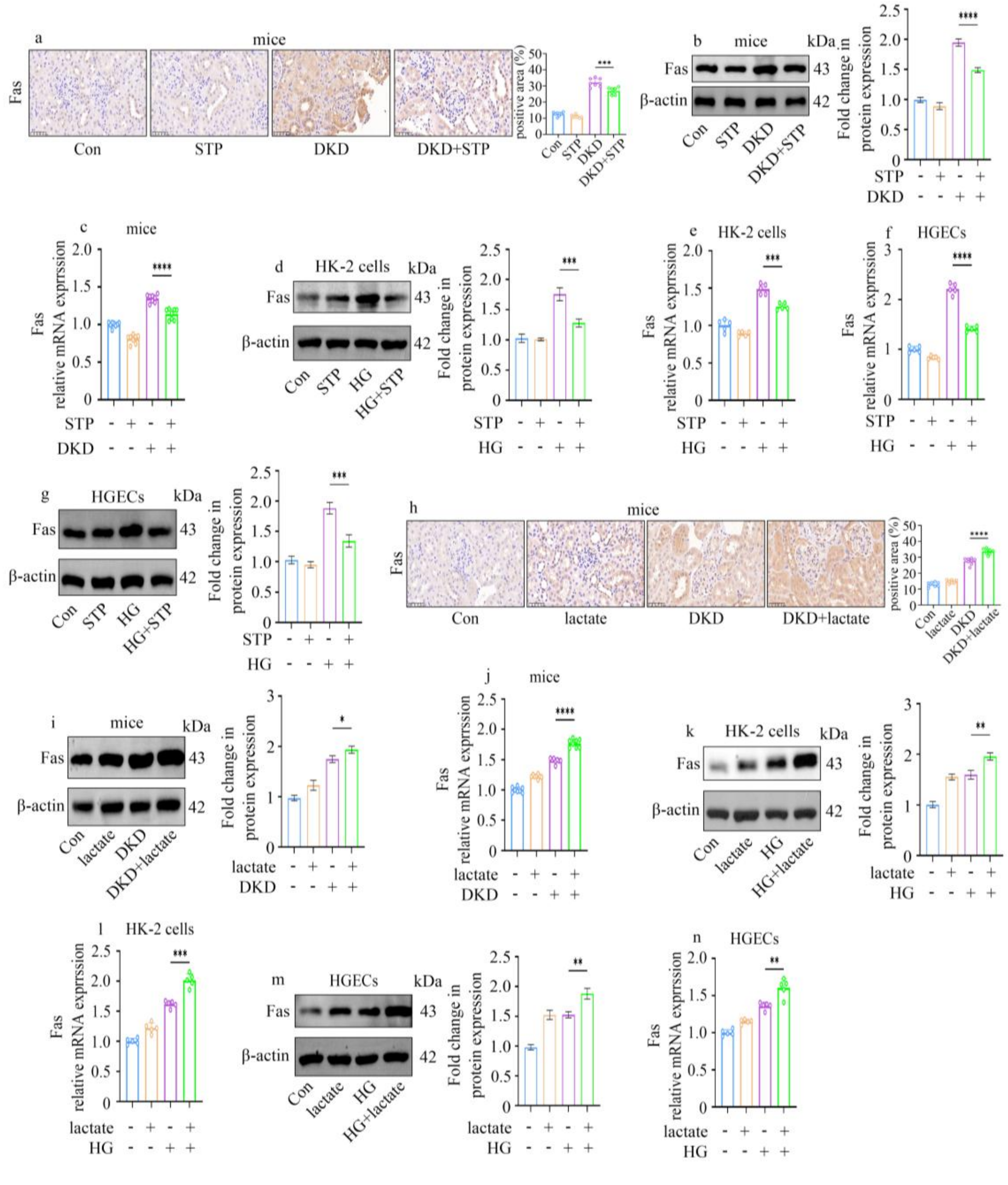

(a) Immunohistochemical (IHC) staining indicated that STP treatment decreased Fas protein expression in the kidneys of DKD model mice (data are presented as the mean ±SD; n=8/group). (b) Western blotting indicated that STP treatment decreased Fas protein expression in the kidneys of DKD model mice. (c) qPCR indicated that STP treatment decreased Fas mRNA levels in the kidneys of DKD model mice (data are

presented as the mean ±SD; n=8/group). (d) Western blotting indicated that STP treatment decreased Fas protein expression in HK-2 cells treated with high concentrations of glucose. (e) The results of the qPCR assay indicated that STP treatment decreased Fas mRNA expression in HK-2 cells treated with high concentrations of glucose (data are presented as the mean ±SD; n=5/group). (f) qPCR indicated that STP treatment decreased Fas mRNA expression in HGECs treated with high concentrations of glucose (data are presented as the mean ±SD; n=5/group). (g) Western blotting indicated that STP treatment decreased Fas protein expression in HGECs treated with high concentrations of glucose. (h) Immunohistochemical (IHC) staining revealed that lactate treatment increased Fas protein expression in the kidneys of DKD model mice (data are presented as the mean ±SD; n=8/group). (i) Western blotting indicated that lactate treatment increased Fas protein expression in the kidneys of DKD model mice. (j) The results of the qPCR assay indicated that lactate treatment increased the Fas mRNA level in the kidneys of DKD model mice (data are presented as the mean ±SD; n=8/group). (k) Western blotting indicated that lactate treatment increased Fas protein expression in HK-2 cells treated with high concentrations of glucose. (l) qPCR indicated that lactate treatment increased Fas mRNA expression in HK-2 cells treated with high concentrations of glucose (data are presented as the mean ±SD; n=5/group). (m) Western blotting indicated that lactate treatment increased Fas protein expression in HGECs treated with high concentrations of glucose. (n) The results of the qPCR assay indicated that lactate treatment increased Fas mRNA expression in HGECs treated with high concentrations of

glucose (data are presented as the mean ±SD; n=5/group). *P<0.05 and **P<0.01.

Supplementary Fig. 13. Lactate-induced H3K18la and H3K27la expression increases Fas expression in hyperglycemic cells

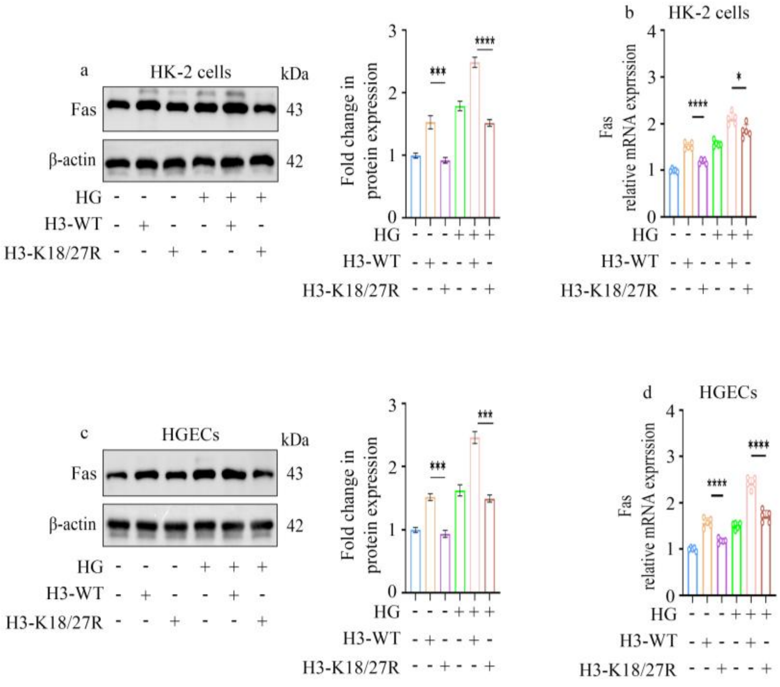

(a) Compared with the H3-WT, the H3-K18/27R mutant significantly inhibited Fas protein expression in HK-2 cells following high-glucose treatment. (b) Compared with the H3-WT, the H3-K18/27R mutant significantly inhibited Fas mRNA expression in HK-2 cells following treatment with high concentrations of glucose (data are presented as the mean ±SD; n=5/group). (c) Compared with the H3-WT, the H3-K18/27R mutant significantly inhibited Fas protein expression in HGECs following treatment with high concentrations of glucose. (d) Compared with the H3-WT, H3-K18/27R mutant significantly inhibited Fas mRNA expression in HGECs following treatment with high concentrations of glucose (data are presented as the mean ±SD; n=5/group). *P<0.05 and **P<0.01.

Supplementary Fig. 14. KRT5 acts as a lactyltransferase that lactylates H3K18 and H3K27

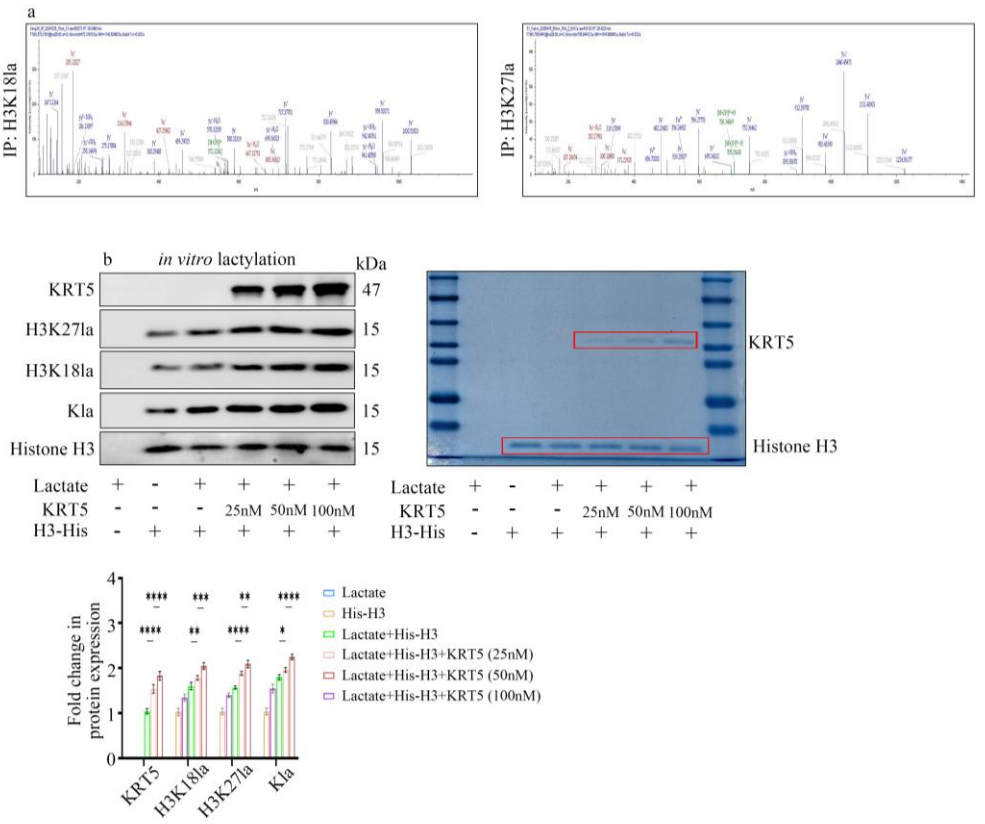

(a) Co-IP coupled with MS analysis was conducted. Our data indicated that KRT5 may be associated with H3K18la and H3K27la. (b) An *in vitro* lactylation assay indicated that KRT5 uses lactate as a substrate to lactylate H3K18 and H3K27 in a concentration-dependent manner.

Supplementary Fig. 15. Expression of KRT18 and KRT5 in diabetic patients and DKD models

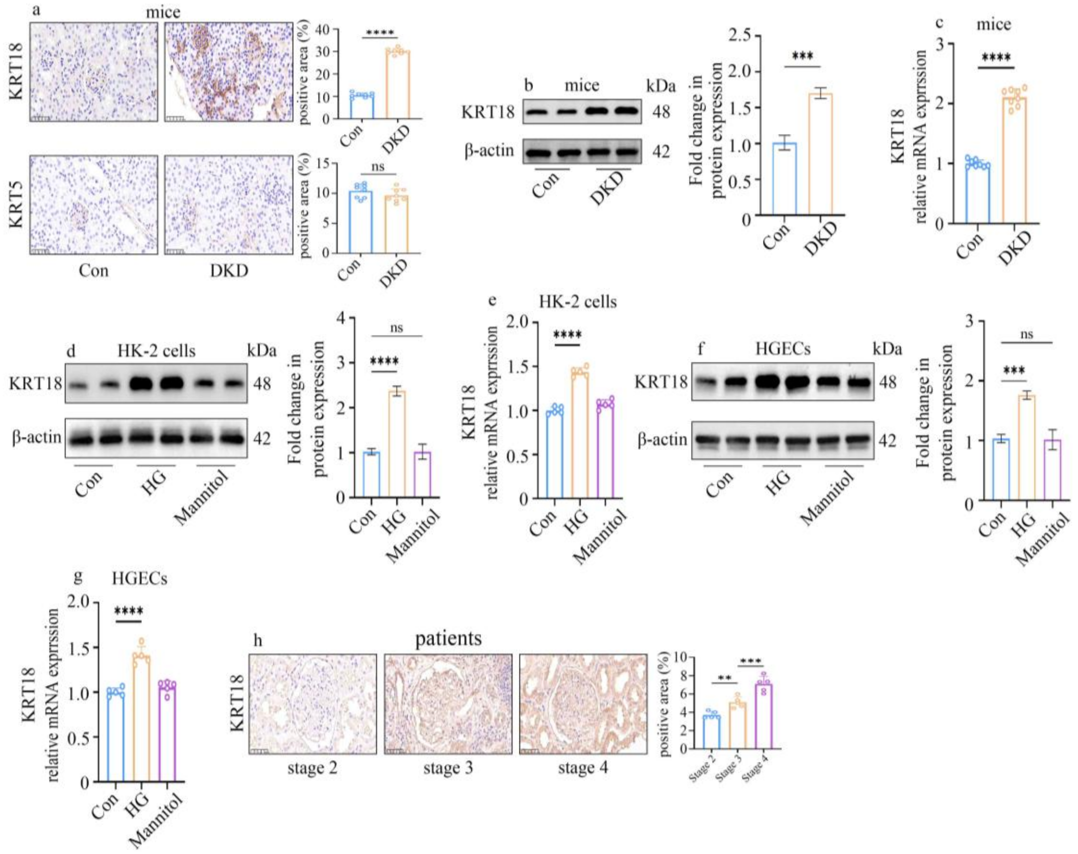

(a) Immunohistochemical (IHC) staining was performed in the present study (scale bar: 50 μm). KRT18 expression was increased in the kidneys of DKD model mice, whereas the KRT5 level did not change (data are presented as the mean ±SD; n=8/group). (b) Western blotting indicated that KRT18 protein expression was increased in the kidneys of DKD model mice. (c) The results of the qPCR assay indicated that KRT18 mRNA expression was increased in the kidneys of DKD model mice (data are presented as the mean ±SD; n=8/group). (d) Western blotting indicated that KRT18 protein expression was increased in HK-2 cells treated with high concentrations of glucose. (e) qPCR results indicating that KRT18 mRNA expression was increased in HK-2 cells treated with high concentrations of glucose (data are

presented as the mean ±SD; n=5/group). (f) Western blotting indicated that KRT18 protein expression was increased in HGECs treated with high concentrations of glucose. (g) qPCR results indicating that KRT18 mRNA expression was increased in HGECs treated with high concentrations of glucose (data are presented as the mean ±SD; n=5/group). (h) IHC staining was performed in the present study (scale bar: 50 μm). The data revealed that the expression of KRT18 in renal biopsy samples from DKD patients gradually increased with increasing DKD stage (data are presented as the mean ±SD; n=5/group). *$P<0.05$ and **$P<0.01$.

Supplementary Fig. 16. KRT18 acts as a lactyltransferase that lactylates H3K18 and H3K27 in HK-2 cells

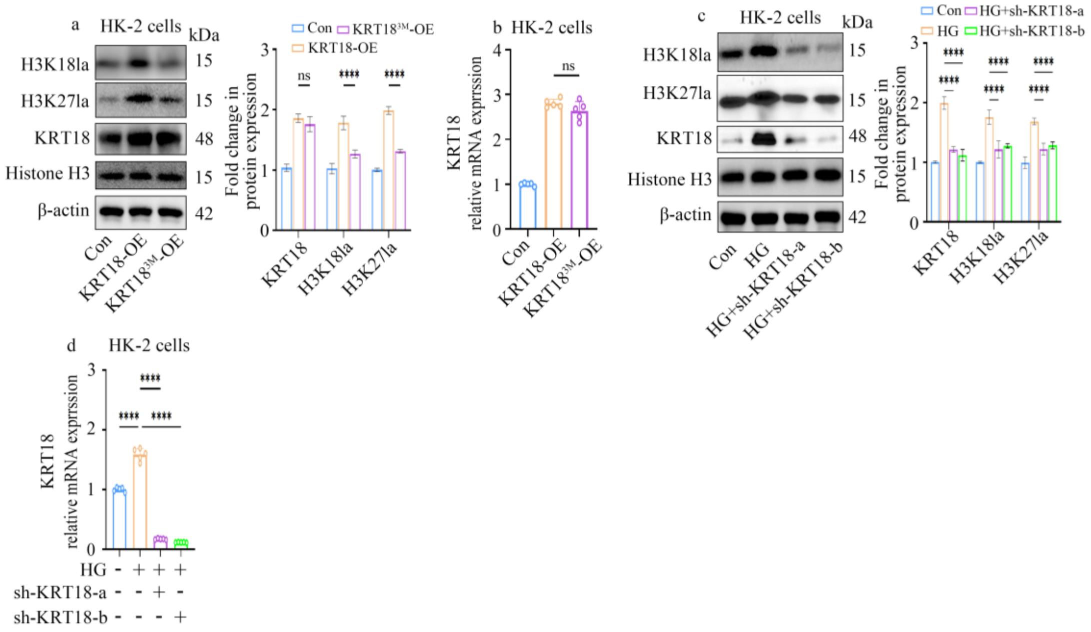

(a) Western blotting revealed that KRT18 overexpression increased H3K18la and H3K27la levels in HK-2 cells. However, the KRT18^{3M} mutant abolished the KRT18-induced increase in H3K18la and H3K27la levels. (b) qPCR assays were performed to measure the KRT18 mRNA levels in HK-2 cells subjected to the corresponding treatments (data are presented as the mean ±SD; n=5/group). (c) Western blotting revealed that KRT18 silencing decreased H3K18la and H3K27la levels in HK-2 cells exposed to high concentrations of glucose. (d) qPCR assays were performed to measure KRT18 mRNA expression levels in HK-2 cells subjected to the corresponding treatments (data are presented as the mean ±SD; n=5/group). *P<0.05 and **P<0.01.

Supplementary Fig. 17. KRT18 participates in high glucose-mediated necroptosis by modulating Fas transcription

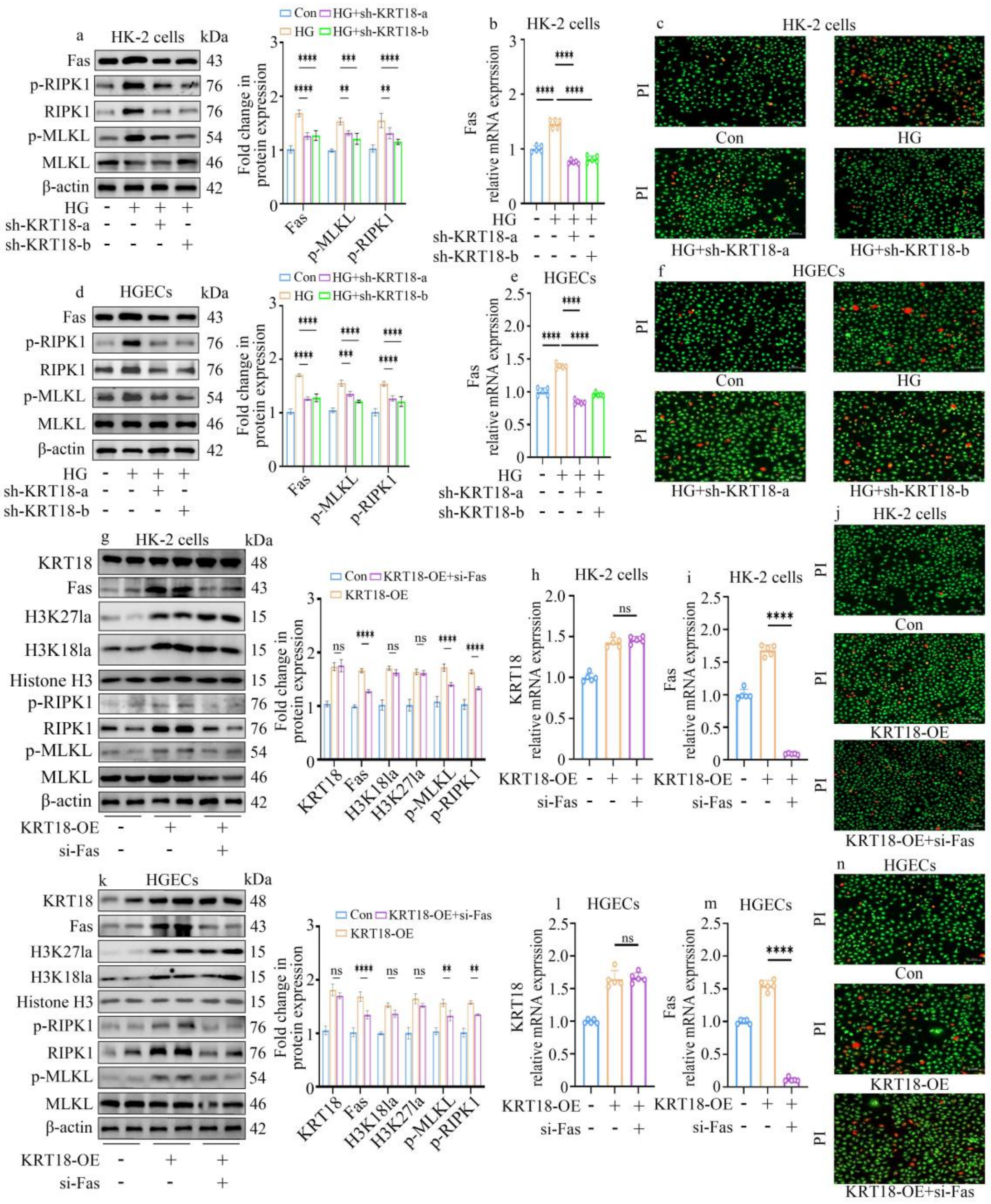

(a) Western blotting indicated that KRT18 silencing reduced Fas, p-RIPK1 and p-MLKL levels in HK-2 cells treated with high concentrations of glucose. (b) The results of the qPCR assay indicated that KRT18 silencing decreased the Fas mRNA level in HK-2 cells treated with high concentrations of glucose (data are presented as

the mean ±SD; n=5/group). (c) KRT18 silencing attenuated the death of HK-2 cells treated with high concentrations of glucose. (d) Western blotting indicated that KRT18 silencing reduced Fas, p-RIPK1 and p-MLKL levels in HGECs treated with high concentrations of glucose. (e) The results of the qPCR assay indicated that KRT18 silencing decreased the Fas mRNA level in HGECs treated with high concentrations of glucose (data are presented as the mean ±SD; n=5/group). (f) KRT18 silencing attenuated the death of HGECs treated with high concentrations of glucose. (g) Western blotting indicated that KRT18 overexpression increased H3K18la and H3K27la levels. Moreover, KRT18 overexpression increased Fas, p-RIPK1 and p-MLKL levels, and these increases were inhibited by Fas silencing in HK-2 cells. (h) qPCR was performed to detect KRT18 mRNA expression (data are presented as the mean ±SD; n=5/group). (i) qPCR indicated that KRT18 overexpression increased Fas mRNA expression in HK-2 cells treated with high concentrations of glucose, but these changes were reversed by Fas silencing (data are presented as the mean ±SD; n=5/group). (j) KRT18 overexpression increased the death of HK-2 cells treated with high concentrations of glucose, and this change was reversed by Fas silencing. (k) Western blotting indicated that KRT18 overexpression increased H3K18la and H3K27la levels. Moreover, KRT18 overexpression increased Fas, p-RIPK1 and p-MLKL levels, and these increases were inhibited by Fas silencing in HGECs. (l) qPCR was performed to detect KRT18 mRNA expression (data are presented as the mean ±SD; n=5/group). (m) qPCR indicated that KRT18 overexpression increased Fas mRNA expression in HGECs treated with high concentrations of glucose, but

these changes were reversed by Fas silencing (data are presented as the mean ±SD; n=5/group). (n) KRT18 overexpression increased the death of HGECs treated with high concentrations of glucose, which was reversed by Fas silencing. *P<0.05 and **P<0.01.

Supplementary Fig. 18. The lactylation activity of KRT18 is involved in the regulation of Fas transcription

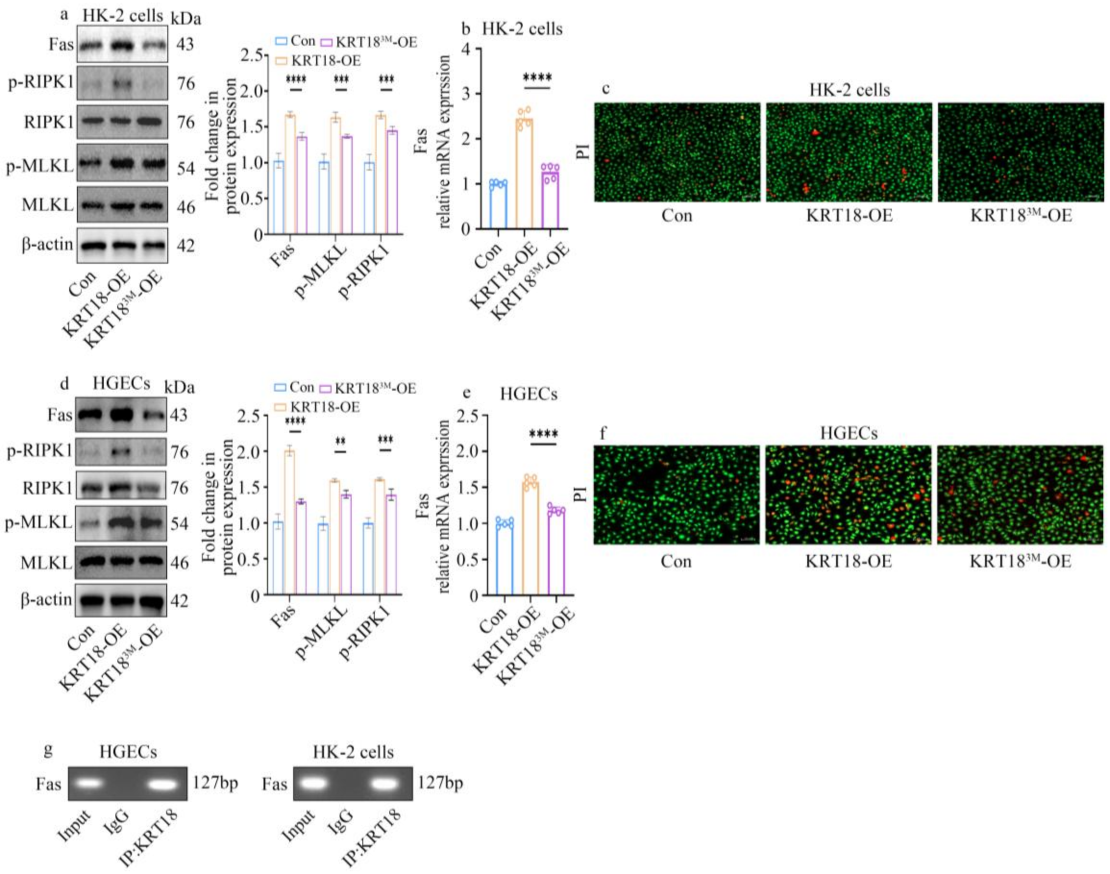

(a) Western blotting indicated that KRT18 overexpression increased the Fas, p-RIPK1 and p-MLKL levels in HK-2 cells, whereas KRT18^{3M} did not affect their levels. (b) The results of the qPCR assay indicated that KRT18 overexpression increased the Fas mRNA level in HK-2 cells, whereas KRT18^{3M} did not have these effects (data are presented as the mean ±SD; n=5/group). (c) KRT18 overexpression increased the death of HK-2 cells, whereas KRT18^{3M} did not have this effect. (d) Western blotting indicated that KRT18 overexpression increased the Fas, p-RIPK1 and p-MLKL levels in HGECs, whereas KRT18^{3M} did not affect their levels (data are presented as the mean ±SD; n=5/group). (e) The results of the qPCR assay indicated that KRT18 overexpression increased the Fas mRNA level in HGECs, whereas KRT18^{3M} did not

have this effect. (f) KRT18 overexpression increased the death of HGECs, whereas $KRT18^{3M}$ did not affect this process. (g) ChIP assays indicated that KRT18 was enriched at the promoter of Fas. *$P<0.05$ and **$P<0.01$.

Supplementary Fig. 19. The lactylation activity of KRT18 is inhibited by treatment with ginsenoside Rc (gRc) *in vitro*

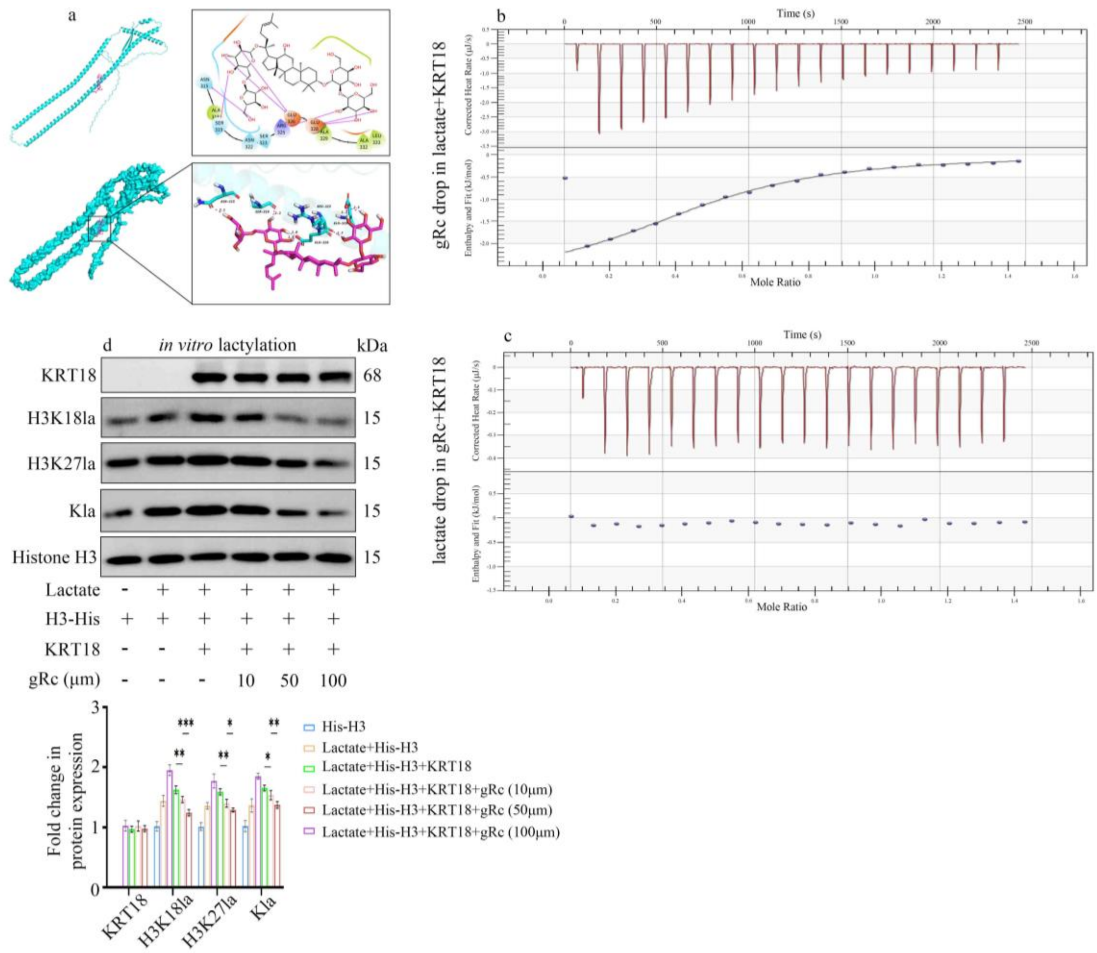

(a) Crystallographic data revealed that gRc interacted with KRT18. (b) During the titration of gRc with the KRT18+lactate mixture, an exothermic event was observed, indicating that gRc is capable of displacing lactate and forming a binding interaction with KRT18. (c) During the titration of lactate with the KRT18+gRc mixture, no exothermic phenomenon was observed, suggesting that lactate was unable to displace gRc from KRT18. (d) An *in vitro* lactylation assay revealed that gRc inhibited the lactyltransferase function of KRT18 in a concentration-dependent manner.

Supplementary Fig. 20. Treatment with ginsenoside Rc (gRc) inhibits the lactylation activity of KRT18 to attenuate necroptosis in hyperglycemic cells

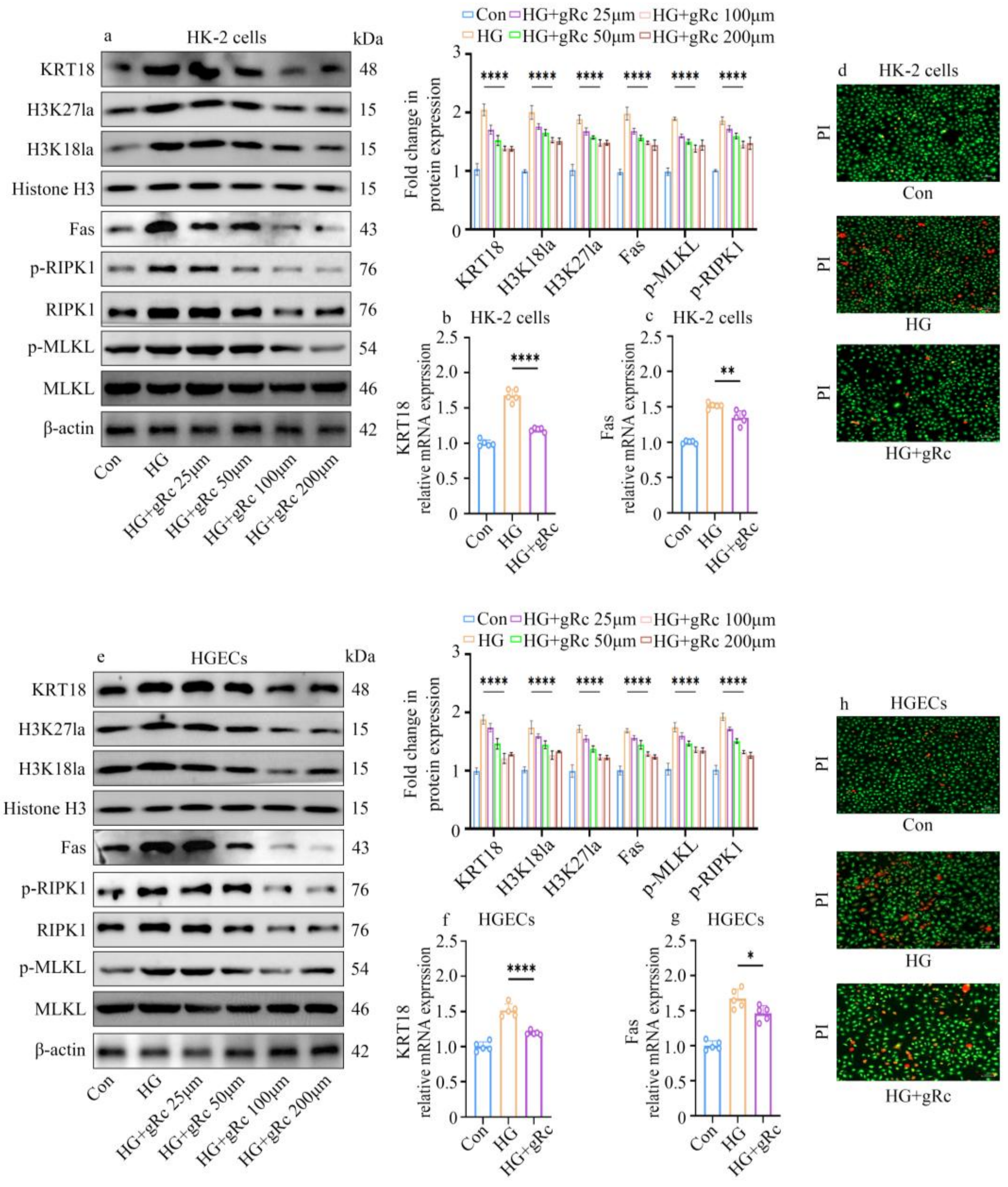

(a) Western blotting showed that gRc treatment decreased KRT18, H3K18la, H3K27la, Fas, p-RIPK1 and p-MLKL levels in a concentration-dependent manner in HK-2 cells treated with high concentrations of glucose. (b) qPCR indicated that gRc treatment decreased the KRT18 mRNA level in HK-2 cells treated with high concentrations of glucose (data are presented as the mean ±SD; n=5/group). (c) qPCR indicated that gRc treatment decreased the Fas mRNA level in HK-2 cells treated with

high concentrations of glucose (data are presented as the mean ±SD; n=5/group). (d) gRc treatment inhibited the death of HK-2 cells treated with high concentrations of glucose. (e) Western blotting revealed that gRc treatment decreased KRT18, H3K18la, H3K27la, Fas, p-RIPK1 and p-MLKL levels in a concentration-dependent manner in HGECs treated with high concentrations of glucose. (f) qPCR indicated that gRc treatment decreased the KRT18 mRNA level in HGECs treated with high concentrations of glucose (data are presented as the mean ±SD; n=5/group). (g) qPCR indicated that gRc treatment decreased the Fas mRNA level in HGECs treated with high concentrations of glucose (data are presented as the mean ±SD; n=5/group). (h) gRc treatment inhibited the death of HGECs treated with high concentrations of glucose. *$P<0.05$ and **$P<0.01$.

Supplementary Tables

Supplementary Table 1. The information of used antibodies

Supplementary Table 2. The sequences of the shRNAs

Supplementary Table 3. Primers used for the real-time RT-PCR analysis

Supplementary Table 4. Primers used for ChIP assay in Fas promoter region

Supplementary Table 5. Serum biochemical indices of the mice

Supplementary Table 6. Metabolomic analysis in DKD mice

Supplementary Table 7. Characteristics of participants diagnosed with diabetic nephropathy

Supplementary Table 8. CUT&Tag data in H3K18la

Supplementary Table 9. CUT&Tag data in H3K27la

Supplementary Table 10. KEGG pathways from H3K18la CUT&Tag data

Supplementary Table 11. KEGG pathways from H3K27la CUT&Tag data

Supplementary Table 12. RNA-seq data from the kidneys of DKD model mice

Supplementary Table 13. The top 50 necroptosis-related genes in GeneCards

Supplementary Table 14. Co-IP coupled with mass spectrometry analysis in H3K18la

Supplementary Table 15. Co-IP coupled with mass spectrometry analysis in H3K27la

Supplementary Table 1. The information of used antibodies

Antibody name	Cat No.	Information
Histone H1 Polyclonal antibody	18201-1-AP	ProteinTech, Wuhan, China
Histone H2A.z Polyclonal antibody	16441-1-AP	ProteinTech, Wuhan, China
Histone H2B Polyclonal antibody	15857-1-AP	ProteinTech, Wuhan, China
Histone H3 Polyclonal antibody	17168-1-AP	ProteinTech, Wuhan, China
Histone H4 Polyclonal antibody	16047-1-AP	ProteinTech, Wuhan, China
Beta Actin Monoclonal antibody	66009-1-Ig	ProteinTech, Wuhan, China
Anti-L-Lactyllysine Rabbit mAb	PTM-1401RM	PTM-BIO, HangZhou, China
Anti-L-Lactyl-Histone H3 (Lys9) Rabbit mAb	PTM-1419RM	PTM-BIO, HangZhou, China
Anti-L-Lactyl-Histone H3 (Lys18) Rabbit mAb	PTM-1406RM	PTM-BIO, HangZhou, China
nti-L-Lactyl-Histone H3 (Lys27) Rabbit pAb	PTM-1428RM	PTM-BIO, HangZhou, China
MLKL Monoclonal antibody	66675-1-Ig	ProteinTech, Wuhan, China
Phospho-MLKL (Ser358) Recombinant antibody	82090-2-RR	ProteinTech, Wuhan, China
RIPK1-Specific Polyclonal antibody	17519-1-AP	ProteinTech, Wuhan, China
Phospho-RIPK1 (Ser161) Monoclonal antibody	66854-1-Ig	ProteinTech, Wuhan, China
LDHA-Specific Polyclonal antibody	19987-1-AP	ProteinTech, Wuhan, China
Cytokeratin 18 Polyclonal antibody	10830-1-AP	ProteinTech, Wuhan, China
Fas/CD95 Polyclonal antibody	13098-1-AP	ProteinTech, Wuhan, China
GST Tag Polyclonal antibody	10000-0-AP	ProteinTech, Wuhan, China

Supplementary Table 2. The sequences of the shRNAs

sh-KRT18-a (human)	CTTCATGAAGAAGAACCACGA
sh-KRT18-b (human)	GATGACACCAATATCACACGA
si-Fas-a (human)	GGAUUGGAAUUGAGGAAGAT
	UCUUCCUCAAUUCCAAUCCTT
si-Fas-b (human)	GGGAAGGAGUACACAGACATT
	UGUCUGUGUACUCCUUCCCTT

Supplementary Table 3. Primers used for the real-time RT-PCR analysis

Human-beta-actin-F-2	CACCATTGGCAATGAGCGGTTC
Human-beta-actin-R-2	AGGTCTTTGCGGATGTCCACGT
Human-LDHA(ID3939)-F	GTGTGCCTGTATGGAGTGGAAT[illegible]
Human-LDHA(ID3939)-R	ATCTTTATCAGTCCCTAAATCTG[illegible]
Human-FAS(ID355)-F2	ACGTCTGTTGCTAGATTATCGTC[illegible]
Human-FAS(ID355)-R2	AGTCTTCCTCAATTCCAATCCC
Human-KRT18(ID3875)-F	ACCATGCAAAGCCTGAACGAC
Human-KRT18(ID3875)-R	CCAAGTGCTCCCGGATTTTG
Mouse-beta-actin--F	CATTGCTGACAGGATGCAGAAG[illegible]
Mouse-beta-actin--R	TGCTGGAAGGTGGACAGTGAGG
Mouse-LDHA(ID16828)-F	AGTTGTTGGGGTTGGTGCTG
Mouse-LDHA(ID16828)-R	TTGGTGTTTTAAGGAAGAGGCT[illegible]
Mouse-FAS(ID14102)-F	TCTCCGAGAGTTTAAAGCTGAG[illegible]
Mouse-FAS(ID14102)-R	GGGCCTCCTTGATATAATCCTTC
Mouse-KRT18(ID16668)-F	TATGAGACAGAACTAGCCATGC[illegible]
Mouse-KRT18(ID16668)-R	CTTCCTTGAGTGCCTCGATTTC
Mouse-Aifm1(ID26926)-F	GGTCCTGATGTCGGCTATGAAG
Mouse-Aifm1(ID26926)-R	CTGTGGCAGATTTTGGGTTGTC
Mouse-Nfkb1(ID18033)-F	GCTGCCAAAGAAGGACACGACA
Mouse-Nfkb1(ID18033)-R	GGCAGGCTATTGCTCATCACAG
Mouse-Ipmk(ID69718)-F	AAACCCTGTATAATGGACGTGA[illegible]
Mouse-Ipmk(ID69718)-R	CTGCTGAATCTTCTCTGATGACG
Mouse-Peli1(ID67245)-F	GCCTTTCCCATACTCCTACTGTG
Mouse-Peli1(ID67245)-R	TACCCATGGTTGCTTTTCATCTA[illegible]

Supplementary Table 4. Primers used for ChIP assay in Fas promoter region

Primer pairs	Sequences
1	F 5’-AAGGATGACTGAAAAGGCCAAGTG-3’<br>R 5’-GTGGAAGATCTGGCAACTCTGAAC-3’

Supplementary Table 5. Serum biochemical indices of the mice

	Con	Lactate	DN	DN+Lactate
FBG(mmol/L)	5.2704±1.0316	5.8648±0.7161	21.421±1.8974$^{\#\#\#\#}$	24.3780±1.0198**
TG(mmol/L)	1.2406±0.0991	1.408±0.1807	2.0266±0.0984$^{\#\#\#\#}$	2.3986±0.2222***
TC(mmol/L)	3.262±0.2436	3.4598±0.4069	4.6838±0.1441$^{\#\#\#\#}$	5.1754±0.313*
BUN(mmol/L)	8.9704±1.0393	10.569±1.4777	16.5876±0.7207$^{\#\#\#\#}$	18.173±0.6282**
HDL(mmol/L)	1.7176±0.1173	1.6002±0.1262	1.0986±0.1653$^{\#\#\#}$	0.8858±0.0406*
LDL(mmol/L)	1.0592±0.1465	1.136±0.1594	1.6446±0.0715$^{\#\#\#\#}$	1.8562±0.0905**
MDA(nmol/ml)	4.1882±0.3588	4.4235±1.2481	6.6106±0.4985$^{\#\#\#\#}$	7.7059±0.5359*
Scr(umol/L)	44.1184±5.2607	48.9368±2.9883	60.8008±3.1829$^{\#\#\#}$	68.9676±3.5004**

	Oxamate	DN+Oxamate	Stripentol	DN+Stripentol
FBG(mmol/L)	5.0352±1.1851	19.0494±1.0264*	5.2674±1.2264	18.7132±1.738*
TG(mmol/L)	1.0524±0.1018	1.7884±0.0934*	1.1156±0.2581	1.7678±0.1159*
TC(mmol/L)	3.013±0.323	4.0332±0.2046**	3.2008±0.217	3.9838±0.1043***
BUN(mmol/L)	8.5836±1.0474	15.1908±0.7898*	8.9622±0.9635	14.8784±0.9189*
HDL(mmol/L)	1.7896±0.1263	1.3278±0.05*	1.7642±0.2058	1.308±0.0796*
LDL(mmol/L)	0.932±0.1012	1.454±0.0746**	1.0426±0.1239	1.4564±0.0833**
MDA(nmol/ml)	4.0118±0.276	5.5271±0.4064**	4.1412±0.7586	5.7694±0.1952**
Scr(umol/L)	43.087±4.5956	54.2278±1.9977**	43.226±5.1173	55.6572±1.9367*

	MLKL$^{-/-}$	DN+MLKL$^{-/-}$	KRT18$^{-/-}$	DN+KRT18$^{-/-}$
FBG(mmol/L)	5.1454±1.7228	18.7086±1.12*	4.9936±1.2866	18.4538±1.1468**
TG(mmol/L)	1.123±0.1485	1.7748±0.1028*	1.1002±0.2398	1.7904±0.1134*
TC(mmol/L)	3.1452±0.3524	4.0362±0.1682**	3.2126±0.1972	4.1348±0.168**
BUN(mmol/L)	8.6234±1.0179	15.2734±0.6739*	8.6818±1.2253	15.2766±0.7813*
HDL(mmol/L)	1.7842±0.1481	1.2894±0.0485*	1.8204±0.1128	1.3958±0.0932**
LDL(mmol/L)	0.9984±0.0785	1.4404±0.085**	0.988±0.0623	1.4636±0.1089*
MDA(nmol/ml)	4.1165±0.6763	5.7482±0.4305*	4.0118±0.8302	5.6353±0.4147**
Scr(umol/L)	42.8038±6.4511	55.4862±3.3332*	42.7866±4.65	56.0896±1.6137*

	Ginsenoside Rc	DN+Ginsenoside Rc
FBG(mmol/L)	5.1368±0.8238	19.0674±1.2477*
TG(mmol/L)	1.0948±0.1461	1.7946±0.1216*
TC(mmol/L)	3.2±0.205	4.1224±0.4477**
BUN(mmol/L)	8.8812±0.8365	15.1078±1.0963*
HDL(mmol/L)	1.7442±0.4413	1.3132±0.1118*
LDL(mmol/L)	1.026±0.0777	1.4548±0.0743**
MDA(nmol/ml)	4.0941±0.7005	5.9176±0.3871*
Scr(umol/L)	43.147±5.219	55.7588±3.1399*

FBG (Fasting Blood Glucose). TG (Triglyceride). TC (Total Cholesterol). BUN (Blood Urea Nitrogen). HDL (High-Density Lipoprotein). LDL (Low-Density Lipoprotein). MDA (Malondialdehyde). Scr (Serum creatinine). Quantitated data were means±SD. Statistical significance was assessed by unpaired t-test (P values adjusted for 5 comparisons); ## P<0.01, ### p<0.001, #### p<0.0001 compared with the Con mouse groups. * p<0.05, ** p<0.01, *** p<0.001, **** p<0.0001 compared with the DN mouse group.

Supplementary Table 7. Characteristics of participants diagnosed with diabetic nephropathy

gender	Age （Year）	BMI (kg/m2)	SBP (mmHg)	DBP (mmHg)	HbA1C (%)	FBG (mmol/l)	CREA (umol/l)	ALB (g/L)	CCr （ml/min ）	24hUPQ （mg）	TP (g/L)	eGFR (ml/min/)
**DN stage 2 （n=5）**												
Male/Female 2/3	59.8±17.2	25.0±4.4	143.0±27.5	78.4±9.0	7.2±1.3	6.9±1.4	93.2±28.1	37.5±6.2	67.8±19.5	2054.2±1179.7	67.0±5.8	68.6±12.2
**DN stage 3 （n=5）**												
Male/Female 5/0	64.8±11.3	24.1±2.4	142.8±16.4	77.4±7.1	9.1±1.6	7.4±3.0	172.1±11.2	32.3±4.3	36.3±8.0	6029.7±4073.0	61.8±4.0	35.7±3.8
**DN stage 4 （n=5）**												
Male/Female 3/2	56.2±18.5	22.7±2.8	144.2±33.6	82.8±20.9	9.0±3.3	10.8±3.2	260.9±36.6	34.8±2.2	22.2±4.6	1809.0±1355.8	60.3±3.1	20.8±3.8

Data are presented as means ± SD. BMI (Body Mass Index), SBP (systolic blood pressure), DBP (diastolic blood pressure), HbA1c (glycated hemoglobin), FBG (fasting blood glucose), CREA (creatinine), ALB (albumin), CCr (Creatinine Clearance), 24hUPQ (24-hour urinary protein quantity), TP (Total Protein), eGFR (Estimated Glomerular Filtration Rate).
